# Land scale division and multifunctional evaluation for Fuping County, China, based on DEM-based watershed analysis

**DOI:** 10.1038/s41598-024-62252-3

**Published:** 2024-05-18

**Authors:** Haikui Yin, Shutao Wang, Yaheng Chen, Yapeng Zhou, Yuqi Chen, Hao Xu

**Affiliations:** 1 College of Resources and Environmental Sciences, Hebei Agricultural University, Baoding, 071001 China; 2 School of Water Conservancy and Hydroelectric Power, Hebei University of Engineering, Handan, 056038 China

**Keywords:** Land, Scale division method, Multifunctional evaluation, County level, Environmental sciences, Environmental social sciences

## Abstract

Land is the spatial background and basic carrier of human survival and development. The study of land function evaluation at different scales can promote the harmonious coexistence of humans and nature. Taking Fuping County, Hebei Province, China, as an example, this study establishes the theoretical framework of county-level land scale division using a digital elevation model (DEM)-based watershed analysis method and establishes the theory and methodological system of land function evaluation from the perspective of the characteristic scale. The multifunctionality of the land was evaluated using the Carnegie–Ames–Stanford approach (CASA), the Integrated Valuation of Ecosystem Services and Trade-offs (InVEST) model and comprehensive index evaluation. By using the methods of DEM-based watershed analysis, dominant factor differentiation and layer superposition, a three-level scale system of ‘subwatershed scale-land chain scale-land segment scale’ and a multifunctional multiscale evaluation index system containing 18 evaluation indices were established. The single-function and multifunction evaluation results of land at different scales were obtained by the comprehensive index method and Getis-Ord Gi* index method. The accuracy of land function evaluation results mainly depends on the selection of the measurement scale. The land measurement scale determined by DEM-based watershed analysis is close to the intrinsic scale of land function evaluation. The scale effect of land function in different temporal and spatial ranges is also evident and shows obvious spatial heterogeneity and difference. At larger scales, individual functions show synergistic effects.

## Introduction

Land is an important component of natural resources and a spatial entity for social and economic development. Driven by two major scientific research programs, namely, the Land Use/Land Cover Change (LUCC) Program^1,2^ and the Global Land Program (GLP)^3^, land science has developed into the core component of global sustainability science and has played an important role since the 1990s^4^. The products and services provided by land are the concrete embodiment of land function. Land function is also the basis for recognizing land systems, exploring land use/cover change, and achieving sustainable utilization of regional resources and sustainable development of the social economy, which directly affects human survival and development^5^. Given the problems of land function dislocation and unreasonable utilization, it is necessary to re-examine the relationship between humans and land from the perspective of land function utilization. Due to the objective existence of the law of natural geographical differentiation, the natural processes and constraints of land change with the choice of research scale, and the same land function may change differently at different scales. The measure used in the study of land function that can reflect its attribute characteristics is the characteristic scale^6^. The scientificity of scale division directly determines the reliability of land function evaluation results. Studying land function evaluation from the perspective of the characteristic scale can reveal the essential attributes and structure of land elements and provide theoretical and methodological references for the multiscale development and multifunctional utilization of land.

The optimization of land spatial patterns needs to be based on the identification and evaluation of land functions^7^. At present, research on land function has focused mainly on its definition and connotation, function classification, and function evaluation. Duan et al.^8^ argued that land function is a comprehensive concept that focuses on the characteristics of all land that actively or passively serves human needs and provides beneficial products. Fei et al.^9^ noted that the three main functions of land production, ecology and life comprise a relationship of unity of opposites and emphasized the internal relations contained in various land functions. Ji et al.^10^ proposed that land functions mainly include production and social and ecological functions. The secondary classification of land function is based on the most important function of the land as the direct division basis and has guiding significance for the establishment of a land function classification system. Based on system theory and Maslow's demand theory, Yang^11^ divided land functions into three first-level categories—ecology, production and society—and 16 s-level categories, 42 third-level categories and 82 fourth-level categories. Paracchini et al.^12^ proposed a further advancement in integrated assessment procedures by setting up an operational multiscale and transparent framework. This framework can be used not only to analyze the possible impact of established policy choices on regional sustainable development but also achieve ex-ante sustainability impact assessment of multifunctional land use under policy constraints.

Xie et al.^13^ constructed a measurement framework model of the multifunctionality of land use in China and determined the corresponding changes in land use functions. They noted that human activities drive spatial and temporal changes in land use, which in turn leads to changes in land use functions. Chen^14^ divided land functions into resource-based functions and ecological functions based on the resources and ecological attributes of land and further subdivided them according to the properties and functional characteristics of land. This author argues that there is currently no science-based land function classification system, mainly because the scale of research has not been unified.

The scale problem is a key issue in the study of land function evaluation^15^. While limitations imposed by natural conditions on land development and utilization have decreased, there is a growing prominence in constraints on human adaptation to resource utilization. Current land use activities suffer from insufficient utilization of land resources and unbalanced development, such as functional dislocation and distortion of characteristic scale selection^16^. Currently, scholars mainly focus on the selection of scales for land function evaluation research, including the administrative region scale, grid scale, and land use type^17^. There are few studies on scale division based on the inherent attributes of land in the study area. Land can be divided according to scales to obtain a hierarchical system that is composed of land units at different levels. The element types and structural composition of the land units at the same scale are basically consistent, and each unit of the scale type is an ideal unit for land function evaluation within the corresponding boundary. The factors that affect land function within the same unit are consistent while the land functions of different land units differ. Thus, relative consistency can be achieved in function evaluation within the same unit. The spatial pattern and processes of land will have different performances at different scales, and the land function also depends on ecological and geographical processes at different spatial and temporal scales^18^. The scientific understanding of land function and its operation pattern requires attention to be paid to t scale effect. At present, there are many macro- and meso-scales, such as climate zone, inter-provincial region, watershed and city, but there are few multifunctional evaluations of small- and medium-sized land, such as county and township^19^. The internal operation mechanism of land under the corresponding scale should be analyzed objectively, and the reasons for the status and performance of land components and their attributes under different spatiotemporal scales should be clarified. The evaluation of land function in different research areas should focus on the selection and differences of different scales. The closer the measurement scale is to the intrinsic scale, the more accurate and reliable the land evaluation results are^20,21^.

In this study, a theoretical analysis framework for land scale division is constructed by means of digital elevation model (DEM)-based watershed analysis, dominant factor analysis and layer superposition space analysis, and an index for the objective evaluation of land function is obtained. From the perspective of the characteristic scale, the characteristics of county-level spatial patterns are analyzed, and the multifunctional evaluation of land is carried out at different scales. This study provides theoretical support and practical reference for promoting the sustainable development of land and improving the management of natural resources.

## Results and discussion

### Scale division analysis results

1. Scale 1: Subwatershed scale

**Figure 1:**
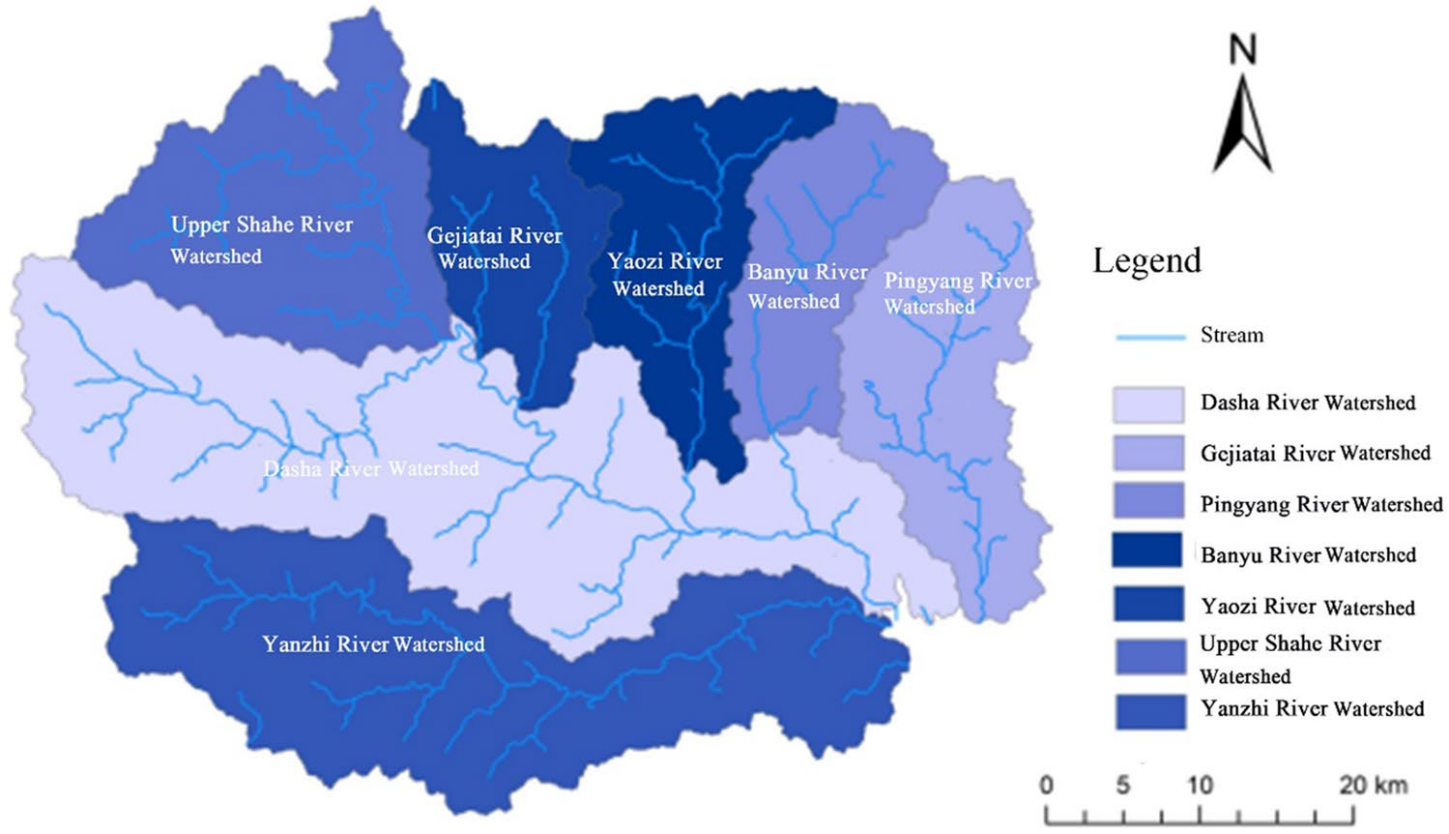
Map of Fuping County at the land chain scale.

The dominant factors influencing the division of subwatersheds include climate, topography, and hydrology. Considering that DEM-based watershed analysis is able to take into account the above influencing factors, which play controlling roles for all each land elements, the subwatershed is used as the first-level scale to divide the land of Fuping County. Each subwatershed is a relatively independent ecological structural unit, and there are obvious differences among the subwatersheds. Based on the ASTER GDEM (resolution, 30 m) dataset (https://www.gscloud.cn/), whose data satisfactorily fits the real river network of Fuping, the threshold for subwatershed delineation was set using the method reported in the literature^22^. Specifically, the threshold of the grid number was set to 500, 800, 1000, 1500, …, and 3000, respectively, the relationship between the river network density and the threshold was fitted and analyzed. Their relationship was in alignment with the power exponent formula y = kx^a^, where *x* is the threshold and *y* is the statisticized digital river network density^22^. The smaller the threshold is, the more the small reiver networks that can be extracted by DEM will be. A power function graph was plotted according to this formula. The boundary point where the river network density changed from slow to stable met the requirement for subwatershed delineation in this study, and a round number of 1000 was determined. Finally, Fuping County was divided into seven subwatersheds, namely, the Gejiatai River Watershed, Banyu River Watershed, Yaozi River Watershed, Pingyang River Watershed, Upper Shahe River Watershed, Yanzhi River Watershed and Dasha River Watershed (Fig. 1).

(2) Scale 2: Land chain scale

**Figure 2:**
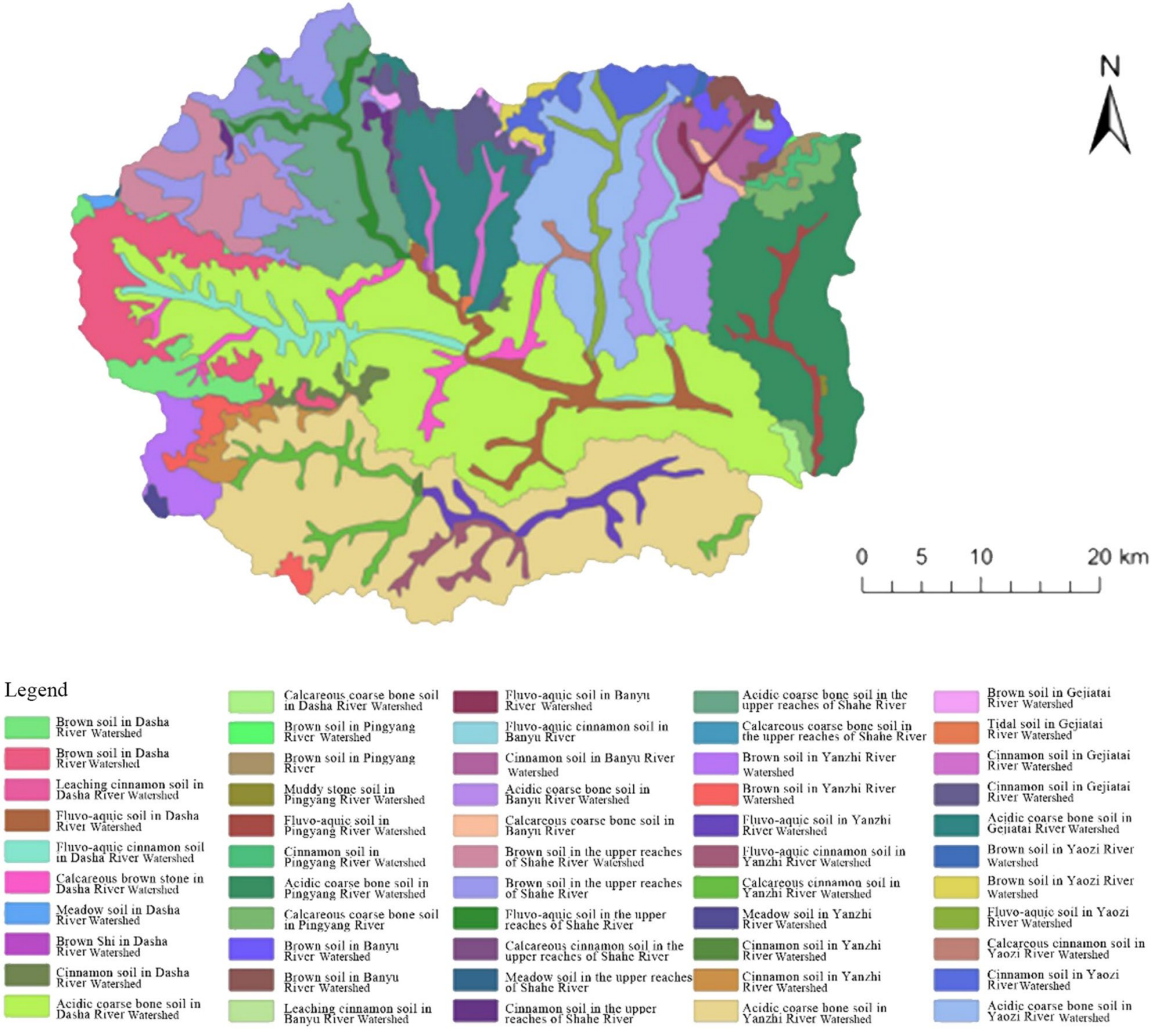
Map of Fuping County at the land segment scale.

The land chain scale is further divided on the basis of the subwatershed scale, which mainly increases the influence of soil elements. Soil is one of the components of land and an important agricultural resource. The distribution of soil types and production capacity directly affect the characteristics and productivity of land resources. Soils can provide water, fertilizer, gas, heat and so on for plant growth and development. In the ‘Chinese Soil Classification System’, the subclass is the continuation of the soil class. The soil type can represent the specific soil formation conditions and dominant soil formation of the soil class. Moreover, additional soil formation processes can be interpreted, and the main attributes of the soil can be comprehensively expressed. Therefore, the soil subclass is taken as the influencing factor of the scale division. At the land chain scale, Fuping County includes 54 specific land type units, such as brown soil in the Dasha River Watershed, calcareous coarse bone soil in the Banyu River Watershed, and brown soil in the Gejiatai River Watershed (Fig. 2). The dominant factors for dividing the land chain include climate, topography, hydrology and soil. These land chains have certain similarities in climate, topography, hydrology and soil properties. At the land chain scale, different land types form a repeated distribution pattern along the terrain profile.

(3) Scale 3: Land segment scale

**Figure 3:**
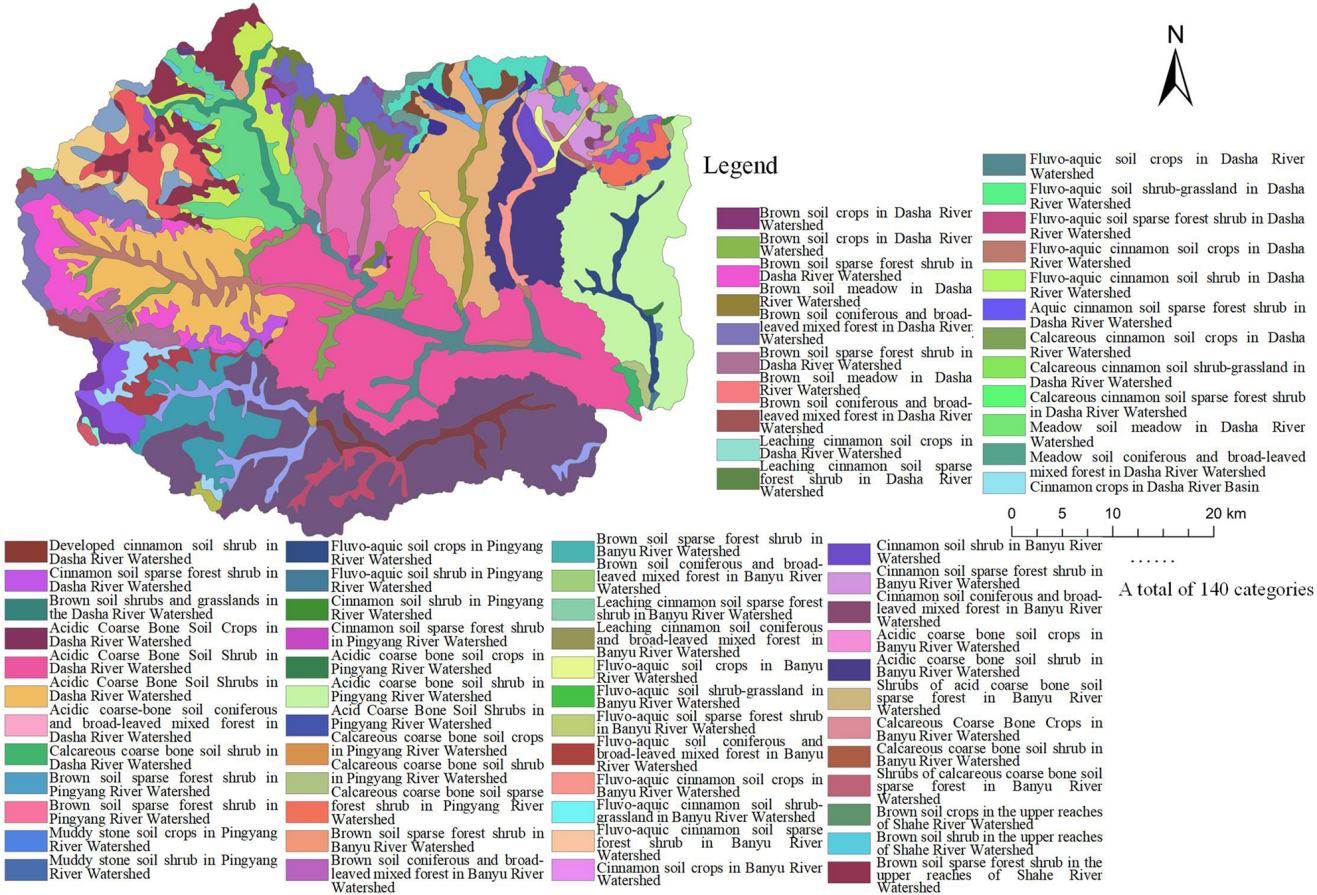
Individual functional evaluation results at the subwatershed scale in Fuping County.

The land segment scale involves further subdivision based on the land chain scale, which increases the influence of vegetation elements. Vegetation is not only the most obvious factor characterizing the landscape but also the most active part of the land ecosystem and plays a leading role in the structure and function of the land ecosystem. Different vegetation types are also the direct causes of landscape diversity. The vegetation types in the Fuping (Yinhe Mountain) Provincial Nature Reserve include cold-temperate coniferous forest, temperate coniferous forest, deciduous broad-leaved forest, mountain birch-poplar forest, deciduous broad-leaved shrub, shrub-grassland, grassland and subalpine meadow. The vertical spectrum of forest vegetation in the jurisdiction is the most representative section of the Taihang Mountains and largely represents the distribution characteristics of forest vegetation in the Taihang Mountains. Changes in the diversity of regional plant types can indicate the succession direction of land types. Similar landscapes composed of land ecosystems, soil types and vegetation types repeatedly occur at the land segment scale. At land segment scale, the land was divided into 140 land type units, including tidal soil shrubs in the Gejiatai River Watershed, meadow soil meadows in the Dasha River Watershed, calcareous coarse bone soil crops in the Pingyang River Watershed, tidal soil coniferous and broad-leaved mixed forests in the Banyu River Watershed, cinnamon soil sparse forest shrubs in the Yaozi River Watershed, tidal soil crops in the upper reaches of the Shahe River Watershed, and acidic coarse bone soil crops in the Yanzhi River Watershed (Fig. 3).

Watershed characteristic parameters are highly important in terms of climate and topography. The land scale division method based on DEM watershed analysis can delineate regional units with independent ecosystem structure, which therefore better represent the comprehensive natural characteristics of land. Scientific land scale division largely depends on the accuracy of measurement scale selection. Currently, a variety of methods are being used to delineate land evaluation units, such as patches and grids, but all these methods have the limitation of imprecise measurement scales. The land scale determined by DEM-based watershed analysis is close to the intrinsic scale for conducting land function evaluation. Liu et al.^23^ constructed a three-level land scales for Heilongjiang Province according to the dimensions of climate impact, geomorphic conditions, geological conditions, soil characteristics and hydrological distribution using the spatial overlaying method based on the GIS platform. In selecting the dominant differentiation factors, they placed emphasis on those which could reflect the characteristics of the natural land conditions as well as on the intrinsic scales of the land. This study was also conducted based on this consideration. The selection of the dominant factors for land scale division based on the intrinsic scale of land is beneficial for eliminating the factors with strong subjective preference, which can make the measurement scale more accurate and thus improve the scientificity of land scale division as well as of the evaluation work following the division.

The land of Fuping County exhibited noticeable spatial heterogeneity at the levels of subwatersheds, land chains and land segments. From the superior scale to the inferior scale, the internal differences of land gradually decreased, while the similarity and homogeneity gradually increased. Li et al.^24^ conducted research on the multi-level classification of the land surface system, with a focus on elucidating the multi-level classification method from block units to the national multi-scale land surface. Although the scope and hierarchy of scale types were comparatively complete, their study had shortcomings in investigating the coupling and coordination relationship between land composition elements at different scales. The scale division method in this study as well as the principles behind the method wase broadly consistent with those in Li et al. study^24^. Through multi-scale land function evaluation, we further clarified the scale effect, environmental gradient, and the variation patterns of the land functions. According to our study, the systematic emergence of land functions is manifested in the non-linear interaction between elements. Based on the results of multifunctional evaluation at the subwatershed scale and the land chain scale, more similar land types appeared at the land segment scale under the joint influence of factors such as watershed, soil type, and vegetation type; in the meantime, with the decrease in the scale, land patches became more specific in spatial distribution, and the spatial distribution of functions also changed from large-scale aggregation to scattered distribution.

### Land multifunctional evaluation results

#### Land multifunctional evaluation method

1. Establishment of the evaluation index system

Clarifying the type of land function is the basis for carrying out multifunctional evaluations of land. Based on the principles of systematicness, dynamics, scientificity, objectivity, accessibility and operability, combined with the existing relevant research results^25,26^ and fully considering the scale effect, the basis of multifunctional evaluation of land, including production function, ecological function, landscape function, raw material supply function, support function and historical record function was determined by the current search team for Fuping County, and based on the sources and characterization factors of the basic components of these land functions, 18 evaluation indices were selected and a multifunctional evaluation index system for land in Fuping County on multiple scales was constructed^27^ (Table 1). Specifically, the vegetation net primary productivity (NPP) value was used as the evaluation indicator of production function, which was calculated using the CASA (Supplementary File for its specific application in this study). For ecological function, water conservation capacity, soil retention, carbon storage and habitat quality were selected using the InVEST model (Supplementary File for its specific application in this study) to evaluate the ecological function of the land spatial continuity. For landscape function, landscape aggregation index was calculated with Fragstats4.2 software while data as to vegetation cover and vegetation type were obtained by analyzing the vector maps of land types in Fuping County. For raw material supply function, soil thickness was obtained through the analysis and extraction of the vector data of the improvement of the quality of cultivated land in Fuping County, soil texture was derived from the soil map of Fuping County, data of the mineral reserves were provided by the survey report on the mineral distribution of Fuping County, and the mineral types were derived by analyzing the geological vector map of Fuping County. For supporting function, slope data were extracted from the DEM data, the types of rock formation groups were determined by analyzing the geological vector map of Fuping County, and groundwater depth was jointly determined by current situation investigation results and data regarding cultivated land quality improvement. For historical record function, the stratigraphic age data were derived from the geological vector map of Fuping County, the soil type was determined by analyzing the soil vector map of Fuping County, and the historical relic data were obtained through a survey of the number of historical relics in each township. To unify the obtained data, the raster data such as NPP, water conservation capacity, soil retention, carbon storage and habitat quality and slope were transformed into vector data and the point data obtained through the analysis of the vector maps were transformed into county-level vector data using Kriging interpolation. All the vector data underwent spatial analysis by ArGIS software and were ultimately unified into vector data with a resolution of 30 m × 30 m.

**Table 1:** Land multifunctional evaluation index system of Fuping County.

Function type	Index	Index attribute	Unit
Production function	Vegetation net primary productivity value	+	gC/(m^2^ a)
Ecological function	Water conservation capacity	+	mm
	Soil retention	+	t/(hm^2^ a)
	Carbon storage	+	Mg
	Habitat quality	+	–
Landscape function	Landscape aggregation index	+	–
	Vegetation cover	+	%
	Vegetation type	+	–
Raw material supply function	Soil thickness	+	cm
	Soil texture	+	–
	Mineral reserves	+	t
	Mineral types	+	–
Supporting function	Slope	−	°
	Rock formation groups	+	–
	Groundwater depth	+	m
Historical record function	Formation age	+	–
	Soil type	+	–
	Historical sites	+	–

(2) Evaluation index processing

1. Standardization of indicators

The dimensions of the evaluation index attributes are not uniform, and they must be treated without quantity before calculation and analysis. At present, there are many methods for standardizing evaluation index attributes, and the hierarchical assignment method and the fuzzy membership function method are more commonly used. The value of the evaluation index attribute should be expressed in quantity as much as possible, and nonnumerical index attributes should be assigned text descriptions. According to expert experience, combined with the influence degree and condition of the index on the function, a score is given to quantify the treatment, and the score system adopts the semiclosed interval or percentage system of [0,1]. By quantifying the processed values, the original data are converted into dimensionless index evaluation values, and functional calculations can be performed.

To make the indicators comparable, this study uses the method of range standardization to nondimensionalize the evaluation indicators. The calculation formula of the positive index is as follows:

1 $$w_{j} = \frac{{d_{j} }}{{\sum\nolimits_{j = 1}^{n} {} }}d_{j}$$

The calculation formula of the negative index is as follows:

2 $$P_{ij} = \frac{{Y_{ij} }}{{\sum\nolimits_{i = 1}^{m} {Y_{ij} } }}$$

where Y_ij_ refers to the standardized value of the evaluation index, X_ij_ refers to the evaluation value of the jth evaluation index on the ith research scale, and max (X_ij_) and min (X_ij_) refer to the maximum and minimum values of the jth evaluation index on the ith research scale, respectively.

(2) Index weight determination

The weight value of the index directly affects the comprehensive evaluation^28^. At present, two methods are used to calculate the weight of an evaluation index: subjective weighting and objective weighting. In this study, the entropy weight method is used to assign weights to the evaluation indices (Table 2) according to objective environmental information, which can ensure the objectivity and authenticity of the weights. The calculation formulas is as follows:

**Table 2:** Weights of the land function evaluation indices in Fuping County.

Function type	Characterization factors	Indicator name	Subwatershed scale	Land chain scale	Land segment scale
Production function	Net primary productivity of vegetation	Net primary productivity	0.0499	0.0329	0.0248
Ecological function	Water conservation	Water conservation capacity	0.0617	0.1096	0.0524
	Soil conservation	Soil retention	0.0903	0.1093	0.1751
	Climate regulation	Carbon storage	0.0499	0.0329	0.0248
	Biodiversity maintenance	Habitat quality	0.0561	0.0305	0.0129
Landscape function	Landscape pattern index	Landscape aggregation index	0.0570	0.0055	0.0173
	Vegetation elements	Vegetation cover	0.0400	0.0219	0.0138
		Vegetation type	0.0379	0.0592	0.0833
Raw material supply function	Soil elements	Soil thickness	0.0406	0.1738	0.2164
		Soil texture	0.0557	0.0241	0.0134
	Topographical features	Mineral reserves	0.0379	0.0592	0.0833
	Geological elements	Mineral types	0.0533	0.0240	0.0228
Supporting function	Topographical features	Slope	0.0360	0.0630	0.0333
	Geological elements	Rock formation groups	0.0557	0.0240	0.0134
	Hydrological elements	Groundwater depth	0.1402	0.0526	0.0063
Historical record function	Geological elements	Formation age	0.0568	0.0177	0.0181
	Soil elements	Soil type	0.0431	0.1006	0.1053
	Humanistic elements	Historical sites	0.0379	0.0592	0.0833

A. Proportion of the ith sample value to the index under the jth index:
  3 $$e_{j} = - k \times \sum\nolimits_{n}^{m} {P_{ij} \ln (P_{ij} ),\quad k = \ln (m)}$$
  where m refers to the number of evaluation units.
B. Entropy of the jth index:
  4 $$Y_{ij} = \frac{{\left( {\max (X_{ij} ) - X_{ij} } \right)}}{{\left( {\max (X_{ij} ) - \min (X_{ij} )} \right)}}$$
  where n refers to the number of evaluation indices.
C. Entropy information redundancy of the jth index:
  5 $$Y_{ij} = \frac{{\left( {X_{ij} - \min (X_{ij} )} \right)}}{{\left( {\max (X_{ij} ) - \min (X_{ij} )} \right)}}$$
D. Weight of the jth index:
  6 $$d_{j} = 1 - e_{j}$$

(3) Land function evaluation model

Land multifunction encompasses the comprehensive benefits of each individual function. The multifunction is measured mainly from the perspective of total amount^29^, which is achieved by the method of standardized sum and the method of comprehensive index sum. The standardization sum method involves summing the standardized subfunction values and regarding each subfunction as a whole; however, this method does not consider the overall perspective of land multifunction. In this study, the comprehensive index sum method is used to evaluate the single-function and multifunction values of land in Fuping County from the perspective of total amount. The calculation formulas are as follows:

7 $$S = \sum\nolimits_{j = 1}^{n} {S_{ij} \cdot W_{j} }$$

where S is the function value of a land function at a certain scale; W_j_ is the weight value of the jth index; S_ij_ is the value of the ith evaluation unit and the jth index; and n is the total number of evaluation indices of a certain land function.

8 $$F = \sum\nolimits_{i = 1}^{n} {S_{i} ,} (i = 1,2, \ldots ,n)$$

where F represents the multifunction value of land at a certain scale, *S*_*i*_ is the ith land function value, and n refers to the total number of land function types.

#### Land single function evaluation results

1. Subwatershed scale

**Figure 4:**
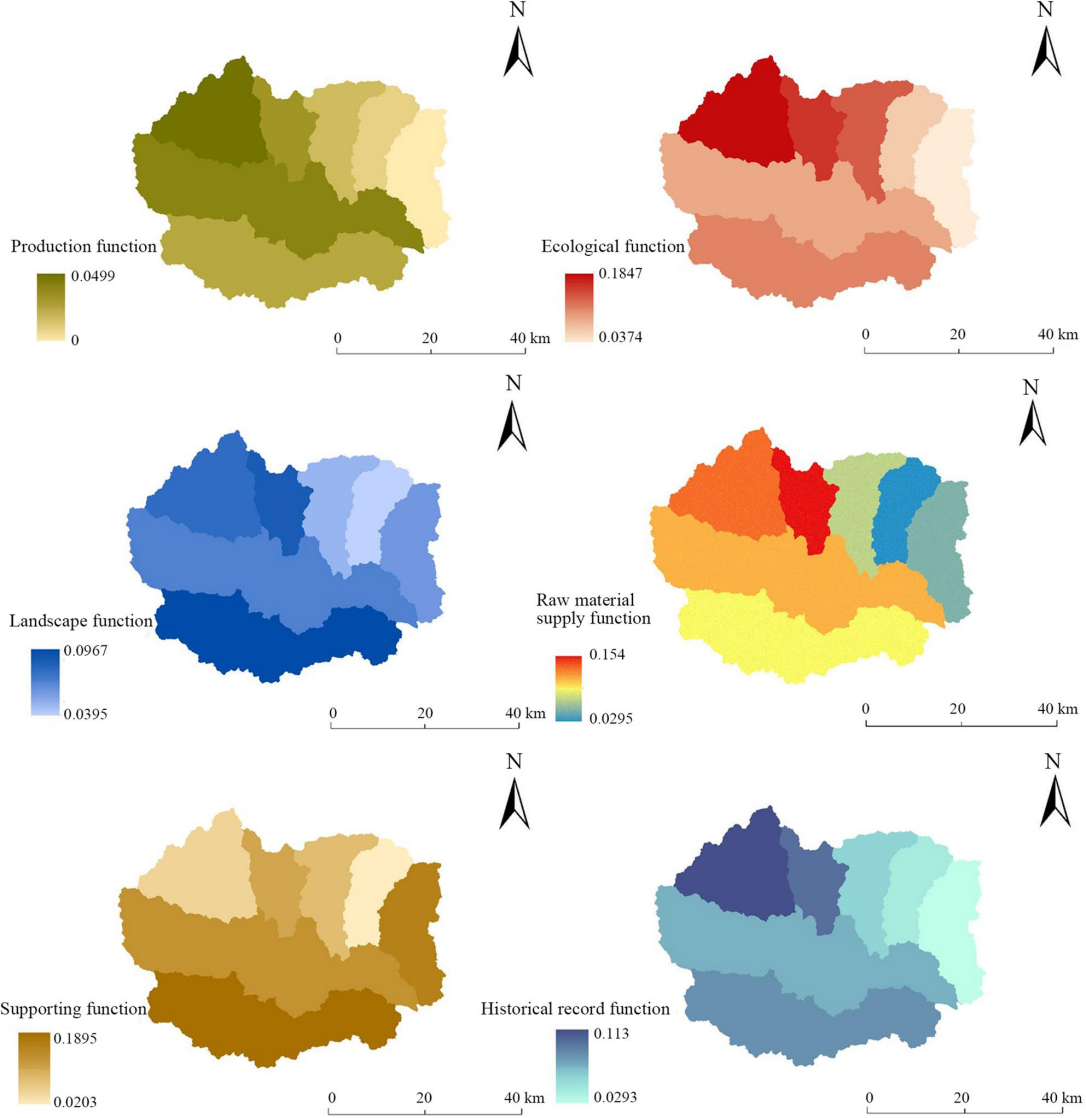
Individual functional evaluation results at the land chain scale in Fuping County.

The production function, ecological function, landscape function, raw material supply function, support function and historical record function of Fuping County were evaluated according to the established multifunctional evaluation index system. The value of each individual function was used as attribute data to construct the functional spatial distribution map (Fig. 4), and the spatial distribution characteristics of each individual function were analyzed.

At the subwatershed scale, the land production function value of Fuping County ranges from 0 to 0.0499 (Table 3), among which the production function of the upper reaches of the Shahe River Watershed is the largest, followed by the production functions of the Dasha River Watershed and the Gejiatai River Watershed; the production function values of the Yanzhi River Watershed, Yaozi River Watershed and Banyu River Watershed are the next smallest; and the production function value of the Pingyang River Watershed is the overall smallest. Fuping County shows a trend toward a ‘higher production function in the central and western regions and a lower production function in the northeast’. In this study, vegetation net primary productivity (NPP) was selected as an index to evaluate the production function. The NPP was determined by calculating the total amount of organic dry matter produced by green plants in the area and over time. The vegetation types in the upper reaches of the Shahe River Watershed are diverse, the area of sunny mountain slope is large, and the soil fertility is high. Although the basic conditions of agricultural production are not superior, the total amount of vegetation is large, and comprehensive factors determine the higher production function.

**Table 3:** Individual and total functional values at the subwatershed scale.

Name	Production function	Ecological function	Landscape function	Raw material supply function	Supporting function	Historical record function	Total function
Bangyu River watershed	0.0090	0.0750	0.0395	0.0295	0.0203	0.0387	0.2119
Dasha River watershed	0.0320	0.0769	0.0674	0.1194	0.1389	0.0814	0.5160
Gejiatai River watershed	0.0252	0.1716	0.0885	0.1540	0.0612	0.1029	0.6033
Pingyang River watershed	0.0000	0.0374	0.0485	0.0711	0.1774	0.0293	0.3637
Upper Shahe River watershed	0.0499	0.1847	0.0779	0.1196	0.0292	0.1130	0.5742
Yanzhi River watershed	0.0186	0.1047	0.0967	0.1192	0.1895	0.0959	0.6245
Yaozi River watershed	0.0130	0.1351	0.0401	0.0782	0.0600	0.0729	0.3994

The ecological function value at the subwatershed scale ranges from 0.0374 to 0.1847, among which the ecological function value of the upper reaches of the Shahe River is the largest, followed by that of the Gejiatai River Watershed, Yaozi River Watershed and Yanzhi River Watershed, and the ecological function values of the Dasha River Watershed, Banyu River Watershed and Pingyang River Watershed are the smallest. The overall distribution characteristics are ‘high in the north and south, low in the middle and east’. In this study, water conservation, soil retention, carbon storage, habitat quality and other indicators were selected to characterize the ecological function. The upper reaches of the Shahe River Watershed receive more rainfall, have high vegetation cover, abundant vegetation, and good soil retention, and habitat quality is high. Although there is no natural water system in the Gejiatai Watershed and the water conservation capacity is relatively low, the surface runoff generated by rainfall during the flood season has been well utilized, and the reservoirs built can also be used for irrigation during the dry season. The soil thickness is mostly above 45 cm, and the soil retention is good. The habitat threat factors of roads and urban settlements are weak, and the habitat quality is excellent. In the Pingyang River Watershed, the terrain is flat, the town residential land area is large, the rainfall amount is low, and the vegetation types are less diverse; thus, the carbon storage and habitat quality are not high, and the ecological function value is not high.

The landscape function value at the subwatershed scale ranges from 0.0395 to 0.0967, among which the landscape function value of the Yanzhi River Watershed is the largest, followed by that of the Gejiatai River Watershed; the values of upper reaches of the Shahe River Watershed and Dasha River Watershed are in intermediate; and the landscape function values of the Pingyang River Watershed, Yaozi River Watershed and Banyu River Watershed in the northeast are relatively low. The distribution characteristics of ' low and more concentrated to the northeast' are generally presented. The landscape agglomeration index, vegetation cover and vegetation type were selected to characterize landscape function. The elevation of the Yanzhi River Watershed is less than 1600 m. The terrain changes are generally gentle, and the connectivity of land types is relatively high. The vegetation cover of shrub grass, sparse forest shrubs and coniferous broad-leaved forest is high, so the landscape aggregation index is the highest. Most of the areas in the Gejiatai Watershed are mainly middle mountains, the connectivity of the land types is relatively high, and the cover of sparse forest shrubs is high. The land remediation scope of the Banyu River Watershed and the Pingyang River Watershed is relatively wide. The connectivity between natural land types is relatively poor, and the cover of shrub and grass vegetation is relatively low, which leads to low landscape function in this area.

The value of the raw material supply function at the subwatershed scale ranged from 0.0295 to 0.1540. The functional values of the Gejiatai River Watershed and the upper reaches of the Shahe River Watershed were greater, followed by those of the Dasha River Watershed and the Yanzhi River Watershed. The functional values of the Yaozi River Watershed, Pingyang River Watershed and Banyu River Watershed in the northeast were relatively low. The distribution characteristics of 'high in the northwest and low in the northeast' are generally presented. The mineral type, soil thickness, soil texture and vegetation type were selected to characterize the raw material supply function. The Gejiatai River Watershed is rich in mineral types, mainly coal and metals in Wuwangkou township, Shawo township and Shijiazhai. The soil particle composition is good, and the soil texture score is high. The Pingyang River Watershed and the Banyu River Watershed have poor soil texture and scarce stratigraphic mineral types. The mineral reserves in the Pingyang River Watershed vary greatly. The thickness of the soil layer in the Banyu River Watershed is mostly 30 cm or less, so the value of the raw material supply function in this area is low.

The supporting function value at the subwatershed scale ranges from 0.203 to 0.1895. The supporting function values of the Yanzhi River Watershed and Pingyang River Watershed are the largest, followed by those of the Dasha River Watershed and Gejiatai River Watershed, and the supporting function values of the Yaozi River Watershed, Upper Shahe River Watershed and Banyu River Watershed are relatively small. The distribution characteristics of 'stronger in the southeast than northwest' are generally presented. The slope, rock formation group and groundwater depth were selected to characterize the supporting function. Although the altitude of the Yanzhi River Watershed is between 100 and 1600 m, the slope change is relatively gentle. The overall elevation of the Pingyang River Watershed is less than 500 m, and the groundwater depth is greater than that of other watersheds, which indirectly determines the topographic, geomorphic and hydrological elements of the supporting function.

The historical record function value at the subwatershed scale ranges from 0.0293 to 0.1130. The function values of the upper reaches of the Shahe River Watershed and Gejiatai River Watershed are the greatest, followed by those of the Yanzhi River Watershed, Dasha River Watershed and Yaozi River Watershed, and the Banyu River Watershed and Pingyang River Watershed have the smallest values The overall distribution characteristics are 'stronger in the northwest than the northeast'. The stratigraphic age, soil composition and vegetation type were selected as functional indicators for evaluating the historical record function. The geological structure, soil evolution history and historical relics are all have indicators of the historical record. The strata of the upper reaches of the Shahe River Watershed and the Gejiatai River Watershed are mainly of Archean and Proterozoic origin, and the soil types are mainly subalpine meadow soil, brown soil and some coarse bone soil. There are many homestays and ancient villages, so the value of the historical record function is high. The Pingyang River Watershed is the opposite, as the geology is dominated by new formations, the soil type is dominated by coarse bone soil, and the folk culture is not unique.

(2) Land chain scale

**Figure 5:**
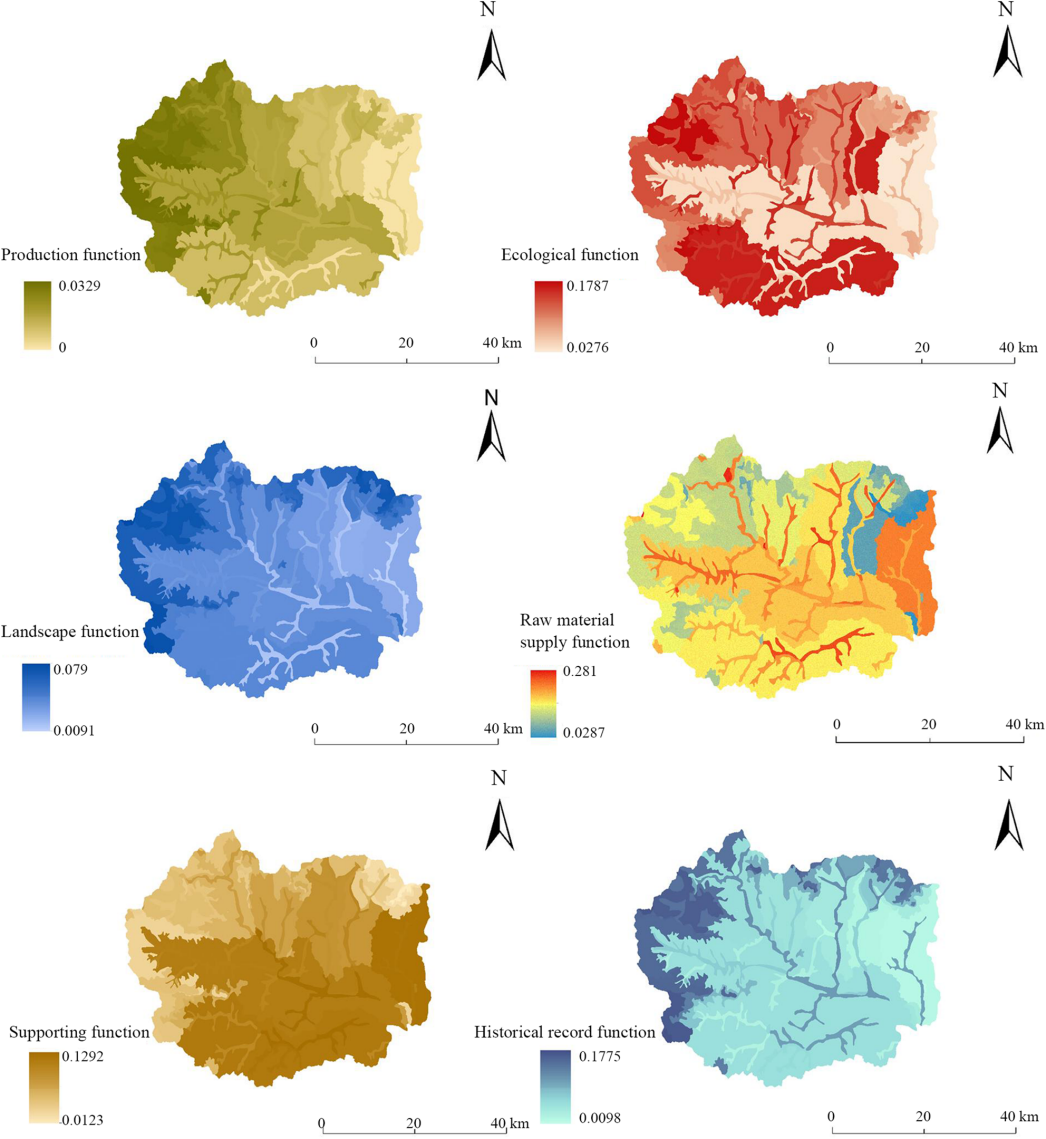
Individual functional evaluation results at the land segment scale in Fuping County.

According to the multifunctional evaluation index system of land, the evaluation results of the production function, ecological function, landscape function, raw material supply function, support function and historical record function at the land chain scale in Fuping County were calculated. The individual function values of 54 evaluation units were used as attribute data (Table 4), and a spatial distribution map of each individual function value was drawn (Fig. 5). Finally, the spatial value distribution characteristics of each individual function at this scale were analyzed.

**Table 4:** Individual and total function values at the land chain scale in Fuping County.

Name	Production function	Ecological function	Landscape function	Raw material supply function	Supporting function	Historical record function	Total function
Fluvo-aquic cinnamon soil in the Banyu River watershed	0.0118	0.0496	0.0140	0.0894	0.0719	0.0577	0.2944
Fluvo-aquic soil in the Banyu River watershed	0.0146	0.0589	0.0301	0.1365	0.0422	0.1032	0.3854
Calcareous coarse bone soil in the Banyu River watershed	0.0139	0.0609	0.0289	0.0287	0.0267	0.0268	0.1858
Cinnamon soil in the Banyu River watershed	0.0131	0.0590	0.0351	0.0665	0.0314	0.0660	0.2711
Leaching cinnamon soil in the Banyu River watershed	0.0107	0.0785	0.0541	0.0515	0.0167	0.0732	0.2849
Acidic coarse bone soil in the Banyu River watershed	0.0088	0.1284	0.0199	0.0398	0.0627	0.0244	0.2840
Brown soil in the Banyu River watershed	0.0076	0.0539	0.0545	0.0471	0.0203	0.1416	0.3249
Brown loamy soil in the Banyu River watershed	0.0091	0.0426	0.0532	0.0448	0.0213	0.1411	0.3123
Meadow soil in the Dasha River watershed	0.0310	0.0724	0.0790	0.0956	0.0306	0.1683	0.4769
Fluvo-aquic cinnamon soil in the Dasha River watershed	0.0217	0.0481	0.0267	0.1355	0.0762	0.0655	0.3737
Fluvo-aquic soil in the Dasha River watershed	0.0140	0.0924	0.0146	0.1082	0.1166	0.0961	0.4420
Calcareous coarse bone soil in the Dasha River watershed	0.0001	0.0557	0.0244	0.0866	0.0593	0.0281	0.2541
Cinnamon soil in the Dasha River watershed	0.0239	0.0465	0.0101	0.2238	0.0681	0.0574	0.4298
Developed cinnamon soil in the Dasha River watershed	0.0271	0.0621	0.0417	0.0606	0.0470	0.0725	0.3111
Leaching cinnamon soil in the Dasha River watershed	0.0329	0.1346	0.0356	0.1757	0.0440	0.0673	0.4900
Calcareous cinnamon soil in the Dasha River watershed	0.0254	0.1105	0.0266	0.1205	0.0721	0.0266	0.3816
Acidic coarse bone soil in the Dasha River watershed	0.0169	0.0390	0.0285	0.0920	0.0828	0.0293	0.2886
Brown soil in the Dasha River watershed	0.0316	0.0776	0.0523	0.0737	0.0387	0.1447	0.4187
Brown loamy soil in the Dasha River watershed	0.0326	0.0840	0.0513	0.0649	0.0300	0.1432	0.4060
Tidal soil in the Gejiatai watershed	0.0221	0.0503	0.0180	0.1380	0.0598	0.0962	0.3844
Cinnamon soil in the Gejiatai watershed	0.0158	0.1435	0.0230	0.1300	0.0637	0.0648	0.4408
Developed cinnamon soil in the Gejiatai watershed	0.0178	0.0970	0.0372	0.0563	0.0417	0.0743	0.3243
Acidic coarse bone soil in the Gejiatai watershed	0.0162	0.0779	0.0285	0.0714	0.0523	0.0292	0.2756
Brown loamy soil in the Gejiatai watershed	0.0187	0.0462	0.0448	0.0665	0.0359	0.1451	0.3572
Fluvo-aquic soil in Pingyang River watershed	0.0104	0.0528	0.0096	0.1073	0.0937	0.0906	0.3644
Calcareous coarse bone soil in the Pingyang River watershed	0.0112	0.0542	0.0353	0.0304	0.0293	0.0270	0.1873
Developed cinnamon soil in the Pingyang River watershed	0.0109	0.0705	0.0375	0.0334	0.0191	0.0667	0.2382
Muddy stone soil in the Pingyang River watershed	0.0000	0.0351	0.0091	0.0467	0.0740	0.0098	0.1747
Acidic coarse bone soil in Pingyang River watershed	0.0053	0.0293	0.0213	0.1270	0.1049	0.0236	0.3115
Brown soil in the Pingyang River watershed	0.0082	0.0792	0.0426	0.0436	0.0179	0.1282	0.3198
Brown loamy soil in the Pingyang River watershed	0.0081	0.0799	0.0460	0.0340	0.0123	0.1353	0.3157
Meadow soil in the upper reaches of the Shahe River watershed	0.0226	0.0276	0.0740	0.2810	0.0529	0.1775	0.6356
Fluvo-aquic soil in the upper reaches of the Shahe River watershed	0.0212	0.1145	0.0240	0.1228	0.0555	0.1016	0.4397
Calcareous coarse bone soil in the upper reaches of the Shahe River watershed	0.0229	0.0425	0.0276	0.2096	0.0436	0.0281	0.3742
Developed cinnamon soil in the upper reaches of the Shahe River watershed	0.0274	0.0657	0.0392	0.0439	0.0322	0.0744	0.2828
Calcareous cinnamon soil in the upper reaches of the Shahe River watershed	0.0193	0.0933	0.0158	0.0480	0.0663	0.0177	0.2605
Acidic coarse bone soil in the upper reaches of the Shahe River watershed	0.0241	0.0779	0.0334	0.0612	0.0421	0.0319	0.2705
Brown soil in the upper reaches of the Shahe River watershed	0.0312	0.1787	0.0547	0.0732	0.0391	0.1497	0.5265
Brown loamy soil in the upper reaches of the Shahe River watershed	0.0294	0.0869	0.0460	0.0616	0.0369	0.1423	0.4030
Meadow soil in the Yanzhi River watershed	0.0226	0.0618	0.0762	0.0993	0.0359	0.1696	0.4655
Fluvo-aquic cinnamon soil in the Yanzhi River watershed	0.0074	0.0458	0.0107	0.1098	0.1010	0.0536	0.3283
Fluvo-aquic soil in the Yanzhi River watershed	0.0060	0.0294	0.0140	0.1632	0.1292	0.0944	0.4363
Cinnamon soil in the Yanzhi River Watershed	0.0177	0.0401	0.0149	0.0500	0.0847	0.0574	0.2647
Developed cinnamon soil in the Yanzhi River watershed	0.0307	0.1493	0.0332	0.0578	0.0432	0.0664	0.3805
Calcareous cinnamon soil in Yanzhi River watershed	0.0197	0.0985	0.0290	0.1047	0.0690	0.0259	0.3468
Acidic coarse bone soil in the Yanzhi River watershed	0.0116	0.1194	0.0297	0.0764	0.0925	0.0293	0.3590
Brown soil in the Yanzhi River watershed	0.0292	0.0645	0.0594	0.0729	0.0366	0.1519	0.4144
Brown loamy soil in the Yanzhi River watershed	0.0295	0.0687	0.0318	0.0574	0.0413	0.1368	0.3656
Fluvo-aquic soil in the Yaozi River watershed	0.0117	0.0947	0.0239	0.1331	0.0657	0.1023	0.4315
Brown loamy soil in the Yaozi River watershed	0.0128	0.0715	0.0425	0.0674	0.0406	0.0779	0.3128
Calcareous cinnamon soil in the Yaozi River watershed	0.0158	0.0563	0.0179	0.1668	0.0734	0.0216	0.3519
Acidic coarse bone soil in the Yaozi River watershed	0.0113	0.0635	0.0252	0.0785	0.0629	0.0279	0.2692
Brown soil in the Yaozi River watershed	0.0086	0.1017	0.0580	0.0382	0.0133	0.1447	0.3644
Brown loamy soil in the Yaozi River watershed	0.0129	0.0795	0.0419	0.0683	0.0433	0.1430	0.3890

Figure 5 shows that the land production function value of Fuping County is in the range of 0–0.0329 at the land chain scale. The production function values of eluvial cinnamon soil in the Dasha River Watershed, brown loamy soil in the Dasha River Watershed, brown soil in the Dasha River Watershed, and brown soil in the upper reaches of the Shahe River Watershed are large. In turn, the production function values of brown loamy soil in the Yaozi River Watershed, developed cinnamon soil in the Banyu River Watershed, calcareous coarse soil in the Banyu River Watershed, and fluvo-aquic soil in the Dasha River Watershed are intermediate. Lastly, the production function values of muddy stone soil in the Pingyang River Watershed, calcareous coarse soil in the Dasha River Watershed, acidic coarse soil in the Pingyang River Watershed, and fluvo-aquic soil in the Yanzhi River Watershed are the small and that of muddy stone soil in the Pingyang River Watershed is minimal. Overall, a trend of 'high production function in the west and low production function in the northeast' is observed. The western part of the brown soil area in the upper reaches of the Shahe River Watershed is mostly subalpine with an elevation of more than 1600 m. The soil fertility is excellent, and the vegetation cover is high, so the NPP is high. In addition to the fluvo-aquic soil on both sides of the Pingyang River, the vegetation types in the other areas of the Pingyang River Watershed are mostly grasses and shrubs, and the quality of organic dry matter that has formed is lower than that in other areas; thus, the production function value is the lowest.

At the land chain scale, the ecological function value of land in Fuping County ranged from 0.0276 to 0.1787. The ecological functions of brown soil in the upper reaches of the Shahe River, developed cinnamon soil in the Yanzhi River Watershed, cinnamon soil in the Gejiatai River Watershed and leached cinnamon soil in the Dasha River Watershed are relatively large. The ecological functions of acid coarse soil in the Yaozi River Watershed, brown soil in the Yanzhi River Watershed, developed cinnamon soil in the upper reaches of the Shahe River Watershed and brown loamy soil in the Yanzhi River Watershed are intermediate. The ecological function values of fluvo-aquic soil in the Yanzhi River Watershed and argillaceous stone soil in the Pingyang River Watershed are low, while that of acid coarse soil in the Pingyang River Watershed is the lowest. The overall trend is ‘high in the south, low in the middle and northeast’. The soil type the upper reaches of the Shahe River Watershed is brown soil, and the vegetation type is mostly trees and shrubs; thus, the NPP is greater. The ecological function of the acidic coarse soil area in the Pingyang River Watershed is closely related to its vegetation type. The carbon storage and habitat quality of the whole Pingyang River Watershed are lower than those of other watersheds, and the ecological function value is low; thus, the NPP value of the acidic coarse soil area is also the smallest.

At the land chain scale, the landscape function values in Fuping County ranged from 0.0091 to 0.0790. The landscape function values of meadow soil in the Dasha River Watershed, meadow soil in the Yanzhi River Watershed, meadow soil in the upper reaches of the Shahe River Watershed and brown soil in the Yanzhi River Watershed are large. The landscape function values of developed cinnamon soil in the Yanzhi River Watershed, brown loamy soil in the Yanzhi River Watershed, fluvo-aquic soil in the Banyu River Watershed and acidic coarse soil in the Yanzhi River Watershed are intermediate. The landscape function values of brown soil in Dasha River Watershed and fluvo-aquic soil in Pingyang River Watershed are small and that of muddy stone soil in Pingyang River Watershed is the smallest. Overall, there is a trend toward ‘high values in the high-elevation areas in the west and north’. The meadow soil in the Dasha River Watershed is located in the alpine meadow area of Waitou Mountain at an altitude of more than 2000 m. The meadow soil has a high landscape aggregation index and good connectivity, so the landscape pattern index is high. The basic rivers and their sides in the muddy stone soil and fluvo-aquic soil areas of the Pingyang River Watershed have few types of vegetation cover and low vegetation cover, which leads to low landscape function.

At the land chain scale, the raw materials supply function values of the land in Fuping County ranged from 0.0287 to 0.2810, among which the function values of cinnamon soil in the Dasha River Watershed, calcareous coarse soil in the upper reaches of the Shahe River Watershed and leaching cinnamon soil in the Dasha River Watershed are large. The supply function values of brown soil in the Dasha River Watershed, brown soil in the upper reaches of the Shahe River Watershed and brown soil in the Yanzhi River Watershed are intermediate. The function values of developed cinnamon soil in the Pingyang River Watershed and calcareous coarse soil in the Pingyang River Watershed are small and that of calcareous coarse soil in the Banyu River Watershed is the smallest. Overall, functional agglomeration is not obvious, and the characteristics of ‘high and low functions concentrated in the Pingyang River Watershed and Banyu River Watershed’ are observed. The stratigraphic age of the cinnamon soil in the Dasha River Watershed and the calcareous coarse bone soil in the upper reaches of the Shahe River Watershed is relatively old. The thicknesses of the soil layer are greater than 45 cm, and there is a long history of soil occurrence, so their raw material supply function values are large. However, there is no specific mineral type in the area of calcareous coarse bone soil in the Banyu River Watershed, the mineral reserves are small, and the thickness of the soil layer is 30 cm or less, so the functional value is low.

At the land chain scale, the supporting function values of land in Fuping County range from 0.0123 to 0.1292, among which the functional values of fluvo-aquic soil in the Dasha River Watershed and acid coarse bone soil in the Pingyang River Watershed are greater than those in the other regions, and the function value of fluvo-aquic soil in the Yanzhi River Watershed is the largest. The functional values of the developed cinnamon soil in the Dasha River Watershed, leached cinnamon soil in the Dasha River Watershed, and calcareous coarse bone soil in the upper reaches of the Shahe River Watershed are intermediate. The supporting function values of leached cinnamon soil in the Banyu River Watershed and brown soil in the Yaozi River Watershed are small and that of brown loamy soil in the Pingyang River Watershed is the smallest. Overall, the area is characterized by a 'high in the southeast and low in the northwest' trend. The area where the fluvo-aquic soil in the Dasha River Watershed is located is flat, and construction land, such as residential areas, is mostly distributed there. The groundwater table is low, the water is easily extracted, and the topography, geomorphology, hydrology and other advantages are obvious, so the supporting function value is large. The brown loamy soil in the Pingyang River Watershed is located in the middle mountain area, which has an elevation greater than 1600 m and steep slopes greater than 25°; thus, the supporting capacity of the land is insufficient.

At the land chain scale, the function values of historical records of land in Fuping County ranges from 0.0098 to 0.1775. The function values of meadow soil in the upper reaches of the Shahe River, brown soil in the upper reaches of the Shahe River and leached cinnamon soil in the Dasha River Watershed are large. The functional values of acidic coarse bone soil in the Yanzhi River Watershed, brown loamy soil in the Gejiatai River Watershed and calcareous cinnamon soil in the Yaozi River Watershed are intermediate. The functional values of calcareous coarse bone soil in the Pingyang River Watershed and Banyu River Watershed are low, while the functional value of the argillaceous stone soil in the Pingyang River Watershed is the lowest. Overall, a trend of ‘high function in the west and low function in other areas’ is observed. The meadow soil in the upper reaches of the Shahe River Watershed and brown soil in the upper reaches of the Shahe River Watershed are located in a region with a long geological history, early soil occurrence and evolution, and rich folk culture; thus, the historical record function is high. The Mesozoic and Cenozoic geological structures of the muddy stone soil in the Pingyang River Watershed are evident, the soil occurrence history is short, and the number of historical sites is small.

(3) Land segment scale

**Figure 6:**
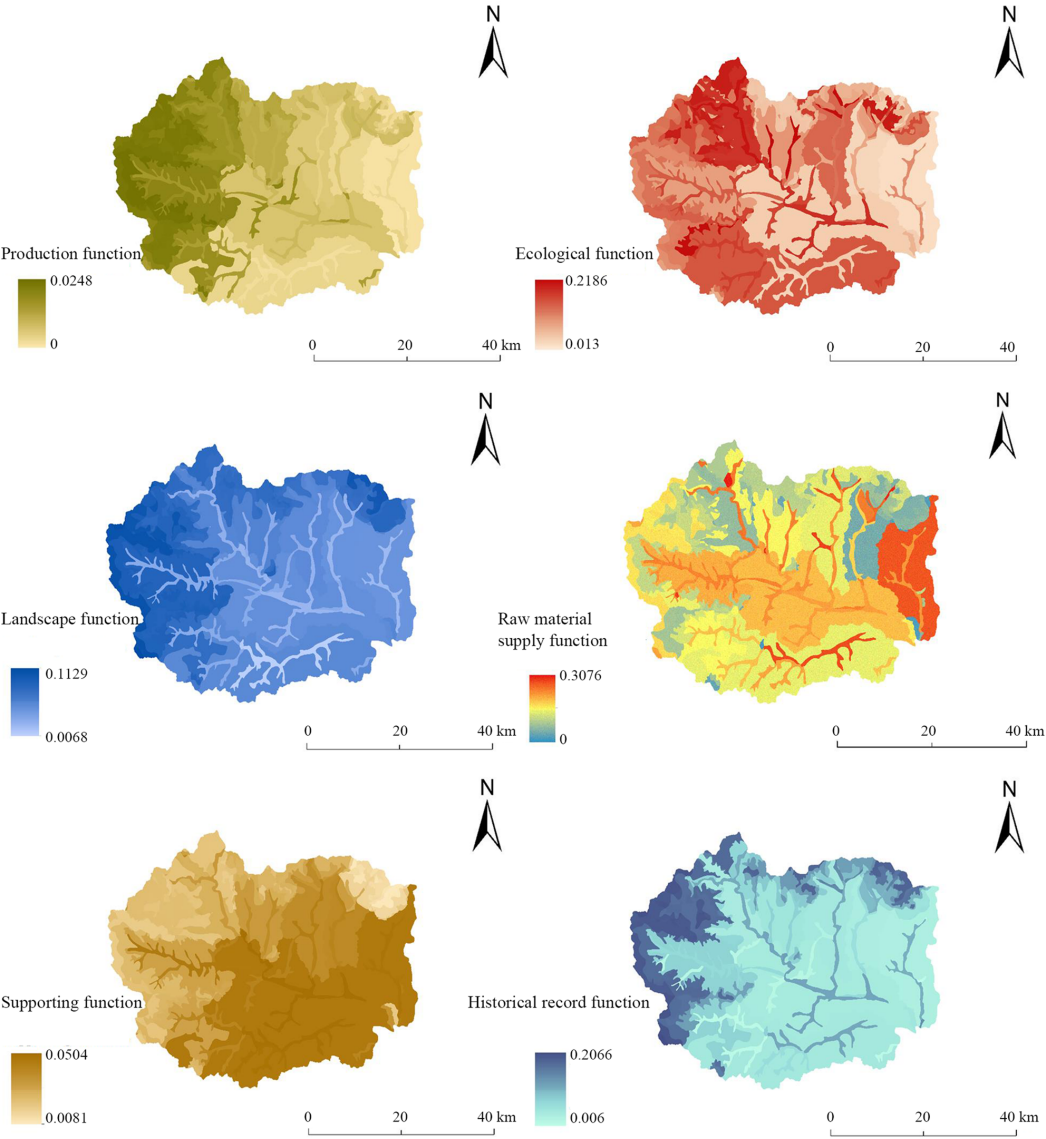
Results of multifunctional evaluation at the subwatershed scale in Fuping County.

The production function, ecological function, landscape function, raw material supply function, supporting function and historical record function of land in Fuping County were evaluated and their values were calculated according to the multifunctional evaluation index system of land. The single function values of 140 evaluation units were taken as attribute data, and a spatial distribution map of each individual function value at this scale was drawn (Fig. 6). Finally, the spatial distribution characteristics of each individual function were analyzed.

The production function values at the land segment scale ranged from 0 to 0.0248, revealing the overall characteristics of ‘high in the northwest and low in the northeast’. There is less construction land in Wuwangkou township in the northwest, and the national highway and expressway does not pass through there; thus, the natural characteristics of the land are relatively well maintained. The altitude of this area is more than 1800 m, and the landform type is mainly a mid-mountain landform. The high vegetation cover in this area has strong soil and water conservation capacity and rich biodiversity, and forest is the main landscape type. The total organic dry matter content of green plants is large, which determines the greater net primary productivity (NPP) value.

The ecological function values at the land segment scale ranged from 0.013 to 0.2186, reflecting the characteristics of ‘prominent in the northwest’ overall. The natural vegetation in this area mainly includes sparse forest, high shrubs, and coniferous and broad-leaved mixed forest. The annual accumulated temperature ≥ 10 °C in this area is greater than 2400 ℃, and the rainfall is sufficient, which provides conditions for good soil fertility, carbon storage and habitat quality; thus, the ecological function of the area is large.

The landscape function values at the land segment scale ranged from 0.0068 to 0.1129, revealing the characteristics of ‘high in the western and northern edges’. This is mainly related to vegetation factors. Coniferous and broad-leaved mixed forests are the main vegetation types. The soil is mainly leached cinnamon soil and brown soil, and a small part of the area is alpine meadow. The area is less disturbed by development and utilization, and the degree of landscape agglomeration is high, so the area has a high landscape function value.

The range of the raw material supply function values at the land segment scale is 0–0.3076, which reflects the characteristics of ‘large area concentrated in the Pingyang River Watershed’. Although the supply of raw materials in some areas of Wuwangkou township and Chengnanzhuang town is relatively high, a large-scale concentration of raw materials is mainly found in Pingyang town and Taiyu township on the east side of the Fuping Plateau. This shows that the choice of different scales will lead to different performances of land components. The area has flat terrain, a small slope and abundant mineral types. The mineral reserves are relatively large and concentrated, which results in a high raw material supply.

The range of supporting function values at the land segment scale is 0.0081–0.0504, which shows the characteristic of ‘low in the northwest and high in the southeast’ overall. The terrain of Fuping County gradually increases from southeast to northwest, the undulation degree increases from 0 to 272°, and the slope increases from 0° to the highest value of 75°. The geological elements in the eastern region are relatively stable, the slope and undulation change gradually, the mechanical properties of the land are generally good, and the land structure, such as geology and hydrology, is also relatively stable and in good order. This good land supporting capacity has prompted Fuping town to develop eastward.

At the land segment scale in the Fuping area, the historical record function values range from 0.006–0.2066, revealing the characteristics of ‘outstanding at 1800 m altitude’ overall. The area above 1800 m in elevation in the Fuping area is mainly distributed in the western and northern middle mountains. The Fuping rock group and its regional metamorphic age are mainly Neoarchean geology. The vegetation is dominated by coniferous broad-leaved mixed forests, while traditional villages and Xiangxian culture areas are more common at higher altitudes. The soil types in the Fuping area mainly include brown soil, brown loamy soil and meadow soil with high fertility. The characteristics of these soils make indicators such as stratigraphic age, soil composition and vegetation type prominent, so they have a high historical record function.

#### Land multifunctional evaluation results

**Figure 7:**
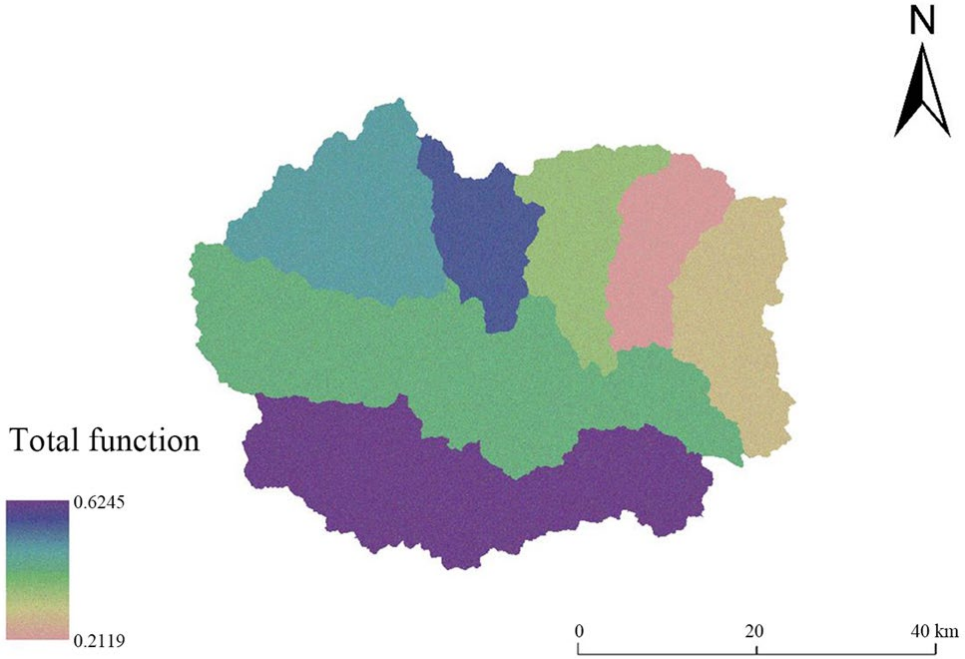
Results of multifunctional evaluation at the land chain scale in Fuping County.

In this study, the comprehensive index method is used to measure the multifunctional values of land at different scales from the perspective of total amount (Fig. 7). The total function value range at the subwatershed scale is 0.2199–0.6245. At the subwatershed scale, the multifunctional values are ordered Yanzhi River Watershed > Gejiatai River Watershed > Shahe Upstream Watershed > Dasha River Watershed > Yaozi River Watershed > Pingyang River Watershed > Banyu River Watershed, revealing the characteristics of ‘higher in the south and west than in the northeast’. The total function values of the Yanzhi River Watershed and Gejiatai River Watershed are high, those of the upper reaches of the Shahe River Watershed and Dasha River Watershed are intermediate, and those of the Yaozi River Watershed, Pingyang River Watershed and Banyu River Watershed are the next lowest. The total function is the sum of the six land functions at each subwatershed and comprehensively reflects the distribution characteristics of multiple land functions across the whole county. In general, the land in the Yanzhi River Watershed and Gejiatai River Watershed has the greatest multifunctional potential and is amenable to priority development and utilization.

**Figure 8:**
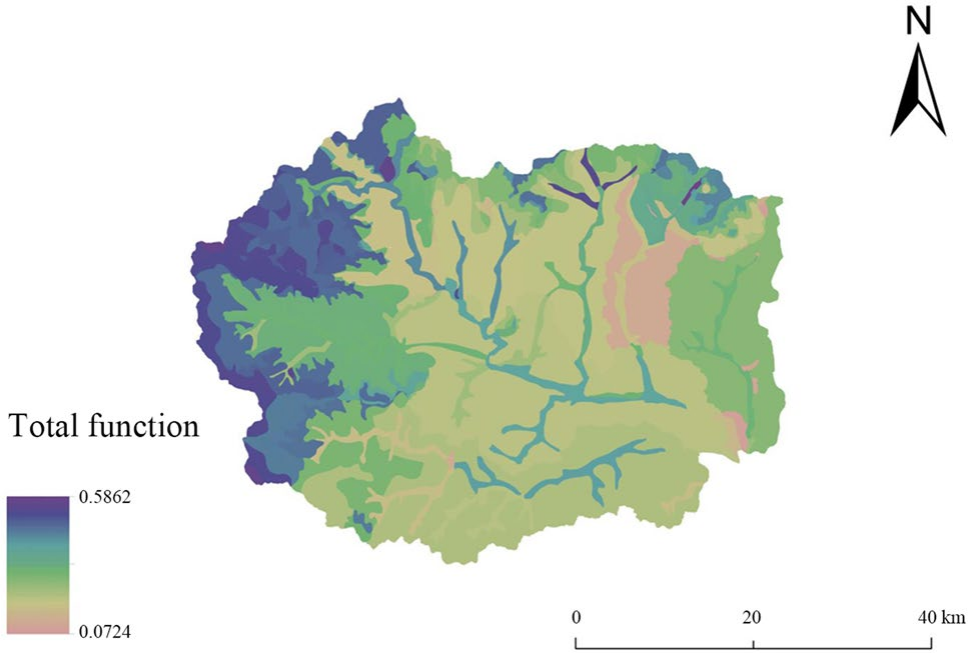
Results of multifunctional evaluation at the land segment scale in Fuping County.

The total function value at the land chain scale ranges from 0.1747 to 0.6356 (Fig. 8). The multifunctional value at this scale shows that the function values of land types such as meadow soil and brown soil in the upper reaches of the Shahe River Watershed and leached cinnamon soil in the Dasha River Watershed are higher. The function values of calcareous coarse bone soil in the Pingyang River Watershed and Banyu River Watershed, and argillaceous stone in the Pingyang River Watershed are lower and show the characteristics of 'higher in the southwest than northeast'. The total function values of meadow soil and brown soil in the upper reaches of the Shahe River, and leached cinnamon soil in the Dasha River Watershed are high. The total function values of acidic coarse bone soil in the Yanzhi River Watershed, brown loamy soil in the Gejiatai River Watershed, and calcareous cinnamon soil in the Yaozi River Watershed are intermediate. The total function values of calcareous coarse bone soil in the Pingyang River Watershed and Banyu River Watershed are low. Compared with the multifunctional evaluation results at the subwatershed scale, the functions at the land chain scale are different, the spatial distribution is more specific, and the function value is positively correlated with soil type, soil quality and vegetation cover. In the future, land use development can be implemented in the east and west at the same time under the land chain scale, and the potential of subalpine land in the west can be utilized in multiple functions. Moreover, attention should be given to the disadvantageous land function of the eastern platform of Fuping.

**Figure 9:**
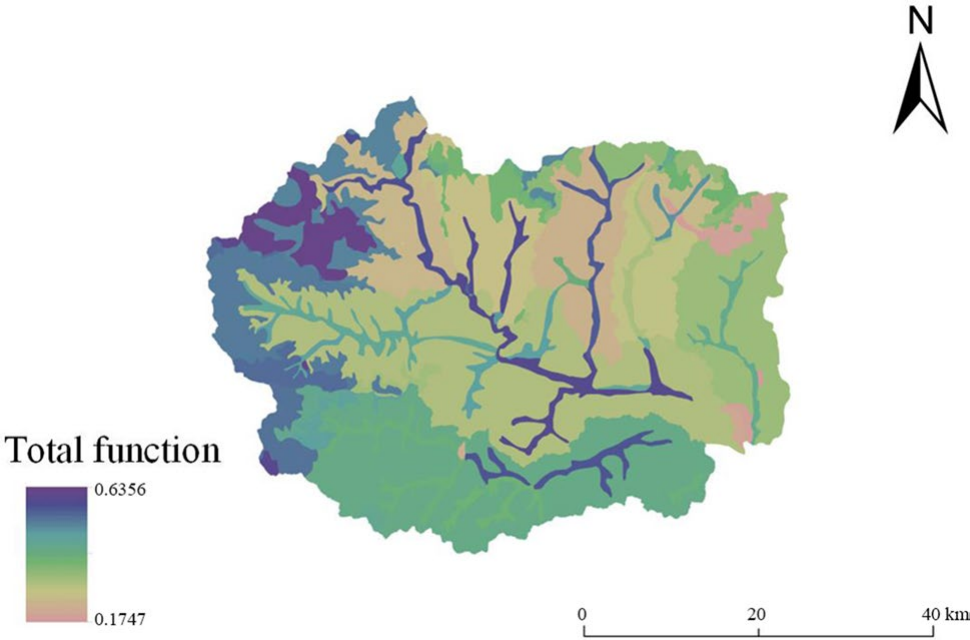
Administrative division map of Fuping County.

The total functional value of the land at the land chain scale ranged from 0.0724 to 0.5862 (Fig. 9). The total functional values of the acidic coarse-grained soil coniferous and broad-leaved mixed forest in the Yanzhi River Watershed, the fluvo-aquic soil sparse forest shrub in the Banyu River Watershed, and the brown soil meadow in the Yanzhi River Watershed were higher. The total functional values of the calcareous coarse-grained soil crops in the Banyu River Watershed and of the argillaceous stone soil crops and calcareous coarse-grained soil crops in the Pingyang River Watershed were lower and showed the characteristics of 'higher in the high-altitude areas in the northwest and lower in the low-altitude areas in the southeast'.

There are scale effects and size effects in the utilization of land resources. The maximum values of multiple functions at different scales followed the order: land chain scale > subwatershed scale > land segment scale. At the land segment scale, there are differences in five indicators, namely, climate, topography, soil type, vegetation type and current utilization mode, which lead to many land types, and the potential of a certain element at individual land types is more prominent. The relationships between different land types are limited, and the size effect is more restrictive. At the land chain scale, there are many similar land types in terms of climate, topography, soil type and so on, which can create better conditions for the synergistic effects of individual functions and comprehensive coordination of land functions at various scales.

Although the spatial distributions of multiple land functions in Fuping County are similar at different scales, different measurement scales will cause spatial differences. At the subwatershed, land chain and land segment scales, the spatial distribution patterns of the production, supporting and historical record functions are similar. However, with the change from the subwatershed to the land chain scale and then to the land segment scale, the nonlinear relationships between the ecological, landscape and the raw material supply functions are highlighted, which leads to different spatial distribution patterns and trends.

In this study, a total of 18 land multifunctional evaluation indices were constructed based on three scales, i.e., subwatersheds, land chains and land segments. The spatial distribution of single function and multifunctional values varied noticeably at different scales, and the functions exhibited certain synergistic effect at the large scale. With Fuping County as the investigated region, Zhao^30^ established a land classification system at the county level using the SOFM model and GIS technology, and furthermore, he applied land type classification results to the practical evaluation of agricultural land quality. Although the scale division system in this study was more complete than Zhao's scale division method, which was based solely on land type, the land multifunctional evaluation results obtained in this study have not yet been applied into research on topics such as land suitability evaluation and comprehensive agricultural zoning nor into practical issues with regard to land spatial use and management such as land consolidation engineering planning and design and farmland quality improvement. Therefore, the practical application effect of the land multifunctional evaluation results obtained in this study from the perspective of characteristic scales should be improved by properly increasing the transitional units of scale division, refining the evaluation units and adjusting the multi-scale evaluation indicator system according to research purpose, which constitute important research direction in the future.

## Conclusion

This study established a theoretical framework for county-level land scale division in China based on the theory, steps and methods of scale division. By relying on DEM-based watershed analysis, a study on the evaluation of land multifunctionality in Fuping County from the perspective of characteristic scales was carried out. The research results provide a theoretical and practical reference for the multiscale development and multifunctional utilization of county land. The following conclusions can be drawn based on this study:

1. Compared with the commonly used administrative scale and grid scale, watershed characteristic parameters can more comprehensively represent the natural characteristics of land in a particular area. The accuracy of the land function evaluation results mainly depends on the accuracy of the selected measurement scale. The land measurement scale determined by DEM-based watershed analysis is closer to the intrinsic scale of land function evaluation. Therefore, studying the spatial pattern of land function in Fuping County from the perspective of characteristic scales is more scientifically sound.
2. The land in Fuping County exhibits obvious spatial heterogeneity at different scales, namely, the subwatershed, land chain and land segment scales. As the scale decreases from large to small, the difference within the land gradually decreases, and the similarity and identity gradually increase. The systematic emergence of land functions is reflected in the nonlinear interaction between elements. Compared with the multifunctional evaluation results at the subwatershed scale and land chain scale, under the combined influence of watershed, soil type, vegetation type and other factors, differences at the land segment scale decrease, while land types with strong similarity become more abundant, and the spatial distribution of land patches becomes more specific. The spatial distribution of the functions also changes from large-scale agglomeration to a scattered distribution.
3. The county land scale division system established in this study can be used to scientifically study the essence of land function and characterize its attribute characteristics. Through the three scales, namely, subwatershed, land chain and land segment, 18 land multifunctional evaluation index systems were constructed, which could reflect the land demand at the characteristic scale, and evaluation indices with practical application were selected. The results of single-function evaluation and multifunction evaluation revealed significant differences in the spatial distribution of function values at the different scales. At large scales, individual functions show synergistic effects.

The scale division method proposed in this article solves the problems of hierarchical structure grading and classification of land systems within county boundaries. Although there are several limitations on a global scale, it is a useful attempt to establish a theoretical framework for global land scale division, which can be supplemented and correlated in theory. In addition, the county land scale type system clearly defines the boundaries of land in different regions, which can be applied to land suitability evaluation and comprehensive agricultural regionalization within a certain range. However, in the context of land remediation projects, cultivated land quality evaluation and other more microscopic land planning projects, it is also necessary to adjust the scale division to adjust the transition unit according to the purpose of the research.

## Data and methods

### Overview of the study area

**Figure 10:**
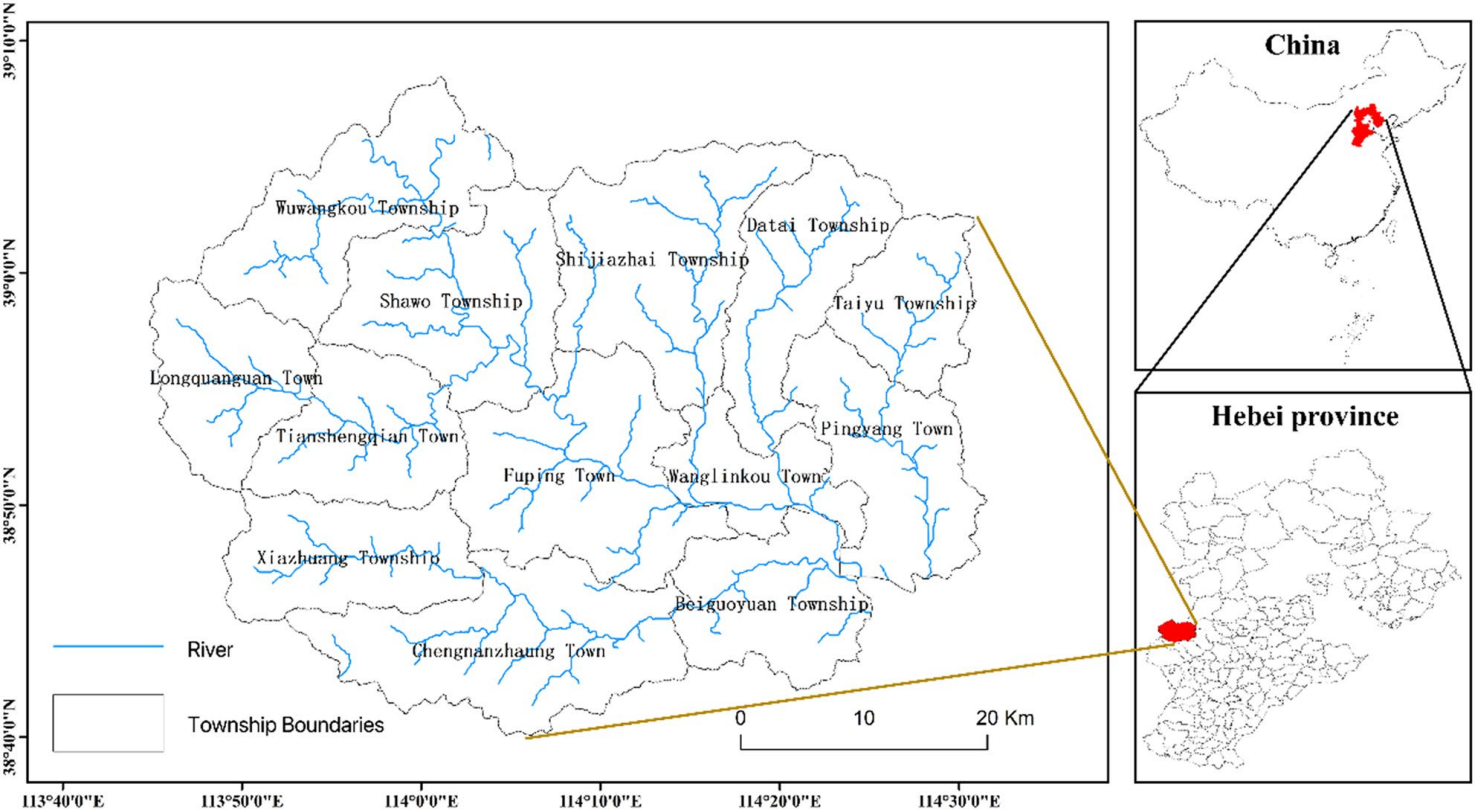
Flowchart of the DEM-based hydrological analysis.

County space is the most evident spatial unit of urban‒rural integration in China and is also the basic unit of economic and social development and administrative management. Considering the actual land management, geographical scope and existing data, this study takes Fuping County as a practical case, and its administrative division map is shown in Fig. 10.

Fuping County is located in the north–central part of the Taihang Mountains in China. It is the junction of Hebei Province and Shanxi Province and is known as the 'throat of Hebei and Shanxi'. The traffic in Fuping County is problematic, and the land conditions are harsh, which the area is referred to as 'nine mountains and half water and half field'. The local industrial base is weak and is part of the concentrated destitute areas of the Taihang Mountains. In 2014, the population of the region exceeded 200,000 people, while the registered poor population was 108,100 people, and the incidence of poverty was as high as 54.37%. The terrain is tilted from northwest to southeast, and the relative height difference between east and west is 2,196 m. A vertical differentiation pattern dominated by altitude is obvious. The geological foundation of Fuping County is extremely old, and its basement rock series belongs to the Archean, including the Fuping Group and the Wutai Group. The region has a temperate semiarid continental monsoon climate, and the difference among the four seasons is considerable. The average annual temperature is approximately 12.6 °C, and the annual temperature difference reaches 30 °C. The precipitation distribution is uneven and is mainly concentrated from June to September, and the annual average precipitation is 547–620 mm. Fuping County is located in the Daqing River system of the Haihe River Watershed and includes the Dasha River, Yanzhi River and other major rivers. In the season of abundant rainfall, the rainwater flows through the valleys of the county, forming a scene of 'nine ditches throughout the county'. These rivers eventually flow into Quyang County and eastward into Baiyangdian Lake. Due to the poor water retention of surface soil, precipitation is mainly lost in the form of surface runoff. There are 6 soil types, 13 subtypes, 35 genera, and 114 species in Fuping County. From the mountaintop to the valley, the soil changes sequentially into subalpine meadow soil, brown soil, coarse bone soil, and tidal soil. Fuping has a wide variety of plant species, including different types of vegetation, such as coniferous trees, broad-leaved trees, shrubs and grasses, and subalpine grasslands. Fuping County governs 8 towns and 5 townships, with a total of 209 administrative villages and 1,208 natural villages. The total area of the county is approximately 2,400 km2, with a total population of 227,700 people. Fuping County has abundant plant resources and agricultural products, among which the main cultivated crops include apple, date, walnut, and potato.

### Data sources

1. Field research. Under the umbrella of the research group, a 3-year field study was conducted from September 2019 to August 2022. During this period, we visited all 13 townships of Fuping County and carried out multiple rounds of surveys and sampling, including interviewing 1285 people from various groups. Through these on-site visits, we determined the soil sampling sites and obtained point data involved in the evaluation index system for land function, such as soil thickness, soil quality, vegetation type, groundwater depth, number of historical sites, and so on. Using the Kriging method^31^, the point data were transformed into surface analysis data.
  We also visited a total of 39 government departments and obtained the vector maps and documents with regard to 1:250,000 scale maps of landform types and soil types, vegetation types and mineral distribution. After data analysis, data of water system distribution, soil type, vegetation type, mineral reserves, mineral types, stratigraphic rock formation and stratigraphic ages were obtained.
2. Meteorological data. From 2021 to 2023, information was obtained from 13 meteorological observation stations in Fuping County, and 10 meteorological monitoring stations were added by the research group according to the altitude gradient and the China Meteorological Data Sharing Service Network. The average values of meteorological data such as precipitation, temperature, accumulated temperature and wind speed were obtained.
3. Vegetation cover data. For vegetation cover, MODIS NDVI data from 2023 were used to crop out the scope of the study area and obtain the normalized difference vegetation index (NDVI). The vegetation types were then confirmed after comparison with field investigation data.
4. Soil data. The data were obtained from the soil type map of Fuping County in 1985, and soil fields were extracted using related tools in ArcGIS. The soil texture, soil thickness, and organic matter content data were provided by the Soil and Fertilizer Station of the Agricultural Bureau of Fuping County or were extracted from the cultivated land quality change database.
5. Topographic and geological data. Topographic data were primarily obtained at the Geospatial Data Cloud website (gscloud.cn), which included ASTER global digital elevation model (GDEM) data and the digital products based on the ASTER GDEM such as elevation, slope, and slope position, at a resolution of 30 m and in a format of raster data. The geological data included stratigraphic rock types and stratigraphic ages. Stratigraphic rock types, such as sedimentary rocks, metamorphic rocks and magmatic rocks, were determined by the hardness of rock texture, and the stratigraphic ages were obtained by analyzing the geological vector maps of Fuping County provided by the local natural resources bureau, which contained the stratigraphic age information including eonothem, erathem, system, series, stage and chronozone.

### Research methods

#### DEM-based hydrological analysis method

**Figure 11:**
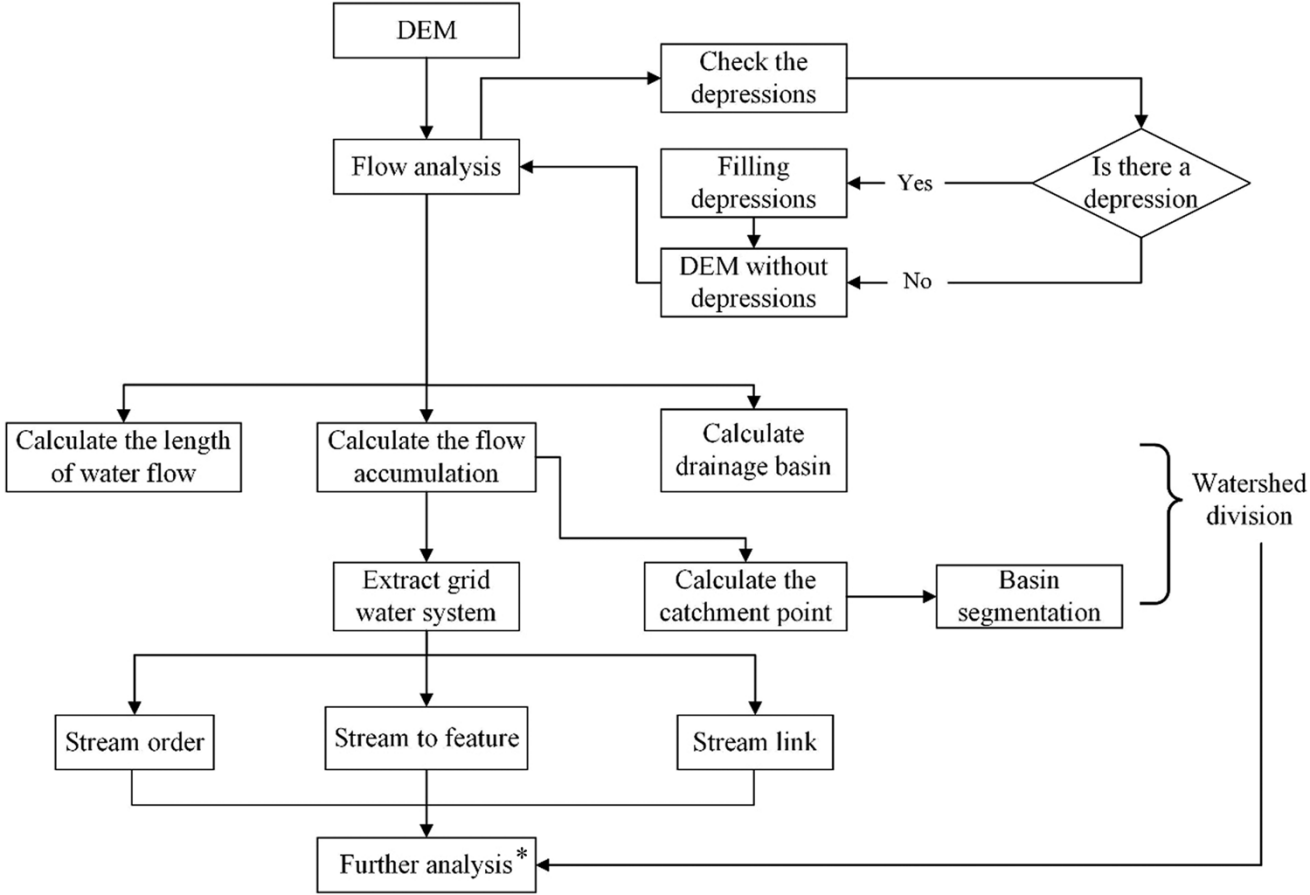
Map of Fuping County at the subwatershed scale. *Refers to the analysis of the comprehensive natural characteristics and spatial differentiation patterns of the land elements at different scales, with the watershed as the land boundary of Fuping.

Hydrological analysis is an important aspect of DEM-based terrain analysis. The hydrological analysis function in ArcGIS software can be applied to extract hydrological characteristic parameters such as flow direction, confluence accumulation and river network, which is highly important in geoscience analysis^32^. The formation of a natural river system is directly affected by watershed characteristic parameters such as the slope, area, channel slope and river network density distribution^33,34^. Therefore, in land planning, agricultural zoning, regional planning and other industries, hydrological analysis is widely used to obtain topographic information and delimit the boundaries of research units^35^. Figure 11 shows the flowchart of the DEM-based hydrological analysis.

#### Mathematical model method

The mathematical models used in this study mainly included the Carnegie–Ames–Stanford approach (CASA) and the Integrated Valuation of Ecosystem Services and Trade-offs (InVEST) model, which are used to reflect the water conservation capacity, soil retention, and carbon storage and habitat quality of the land to protect and improve the environment^36^. These models are also used to calculate the individual values of land functions.

#### Spatial analysis and statistical methods

The ArcGIS 10.2 software platform was used to combine the landscape method and parameter method used in the study of land types and select factors such as altitude, soil type, vegetation type and land use status for spatial analysis. The spatial overlay analysis function of ArcGIS was used to carry out spatial analysis of dominant land factors and establish a land scale type system to describe the spatial attributes and scale effects of land.

### Theoretical framework of scale division

#### Theoretical basis

1. Determination of the land scale based on watershed scope

The range of river control can be indirectly determined by the watershed scope. A watershed is defined as an area covered by rivers, lakes, oceans and other water systems bounded by landforms or a catchment area composed of water systems. The scope of the watershed is based on the catchment area formed by the river or its local area. Moreover, considering the development and utilization of natural resources, a natural geographical unit is also a comprehensive social–economic–political unit and a natural resource utilization and management unit. The watershed unit is a natural flow convergence area of the watershed boundaries in the field of land planning, and it is a land development unit with an independent ecosystem structure^37^. Therefore, the watershed scope is also an ideal research unit for land science^34^. For example, the Yellow River Watershed refers to the area affected by the water system of the Yellow River from the source to the sea. It is an area delineated by the main tributaries of the Yellow River as the backbone and the control area as the scope. It is mainly determined by the characteristics of the water system and topography. The present study takes the Qingshishan red soil subwatershed in Anji County, Zhejiang Province as the basic planning unit, applies GIS technology to the agricultural ecosystem of the subwatershed and carries out land suitability evaluation to ultimately achieve the purpose of effective management and development of red soil resources. In the natural state, the land unit and the watershed unit are spatially coupled. The watershed unit can thus be used and managed based on the distribution, utilization layout and development intensity of the natural land elements to follow the spatial constraints of natural hydrological processes^37^. Therefore, the watershed can be used as the basis for determining the scope of land scale. Compared with the administrative division scale and the grid scale, the watershed can more effectively represent the resource base of the study area. By analyzing watershed characteristics, complex problems related to the utilization of land resources can be solved, such as problems related to natural geographical characteristics, resource investigations and evaluations, water and sediment regulation and safety, watershed environmental protection and land use change simulations^38^.

(2) Land multiscale division based on the theory of land classification

The determination of the watershed scale can be used to define the boundary of a certain scale level of land, while the division of smaller scales needs to further reduce the level of the land system and refine the level of spatial analysis. The core purpose of scale division is to distinguish the internal complexity, that is, the structural level of things. The land scale and spatial analysis level in geography belong to the same category of problems. The scale can indirectly determine the internal complexity of things, the mapping scale and other issues. Different land scales express the complexity of the comprehensive attributes of the elements within a certain region^39^. Generally, from the higher to the lower level of land scale, the internal differences gradually decrease, while the similarity and identity gradually increase.

The multilevel classification of land surface systems is an important method for identifying land systems^40^. Land classification can not only solve the problem of the structural level of land types within a certain scope but also complete the classification of land hierarchical structure, that is, scale type classification. Therefore, through the theory and method of land classification, the division of land scale can be realized, and this approach has certain applicability in the study of land types. In the study of land types, dominant factors are usually used to divide the land level and its individual units, and land type division schemes at different scales are constructed. The key dominant factor classification maps at each scale are analyzed by GIS spatial overlay analysis. On a global scale, land classification relies on the research theories and methods of natural division in physical geography and land types in land science. The land in a certain geographical space contains different levels of individual land units, and many individual land units constitute a hierarchical system. High-level individual units contain low-level individual units. Due to the spatial continuity of the geographical distribution of land, determining the level of individual land units will inevitably be subjective. Therefore, considering the complexity of regional differentiation and to facilitate the comparison and application of the results, it is necessary to determine the criteria for common compliance in the process of land classification.

#### Scale division steps

1. Selection of dominant elements. The selection of the land scale division index can be carried out by quantitative analysis methods such as linear regression and principal component analysis. The research results of land science and soil science studies as well as practical experience in land classification should also be considered^41,42^. The characteristics and nature of land types are often affected by factors such as climate and topography, which are the most significant and decisive factors in a county. According to the land classification theory, the factors that affect the regional differentiation of land generally include soil, vegetation, and current land use. Fuping County is located in the north–central part of the Taihang Mountains. It is a typical mountain structure. The east–west topography is undulating, with a relative height difference of 2196 m, and the vertical differentiation pattern is dominated by altitude. Fuping County has a unique local microclimate, three major rock types, and the soil, vegetation, and current land use types exhibit obvious vertical band spectrum trends. The combination of horizontal zonality and landform is the basic factor influencing the regional differentiation of land. The combination of local climate, vertical zonality and medium landforms is the leading factor of differentiation, and lithology and small landforms are the direct factors of differentiation. In addition, human activities also have an important impact on land differentiation^43^. On the basis of previous research results and combined with the team’s practical multi-year research results from Fuping, climate, topography, hydrology, soil, vegetation and other factors were selected in this study as the dominant differentiation factors of land scale division in Fuping County.
2. Determination of the watershed boundary. Due to the rich water system in Fuping County, the surface runoff in the county shows certain patterns and characteristics, which are directly affected by watershed characteristic parameters such as watershed slope, area, river channel slope and river network density distribution^44^. The characteristic watershed parameters are the cross products of hydrology and geomorphology. The morphological characteristics and topographic relief around the watershed are the direct embodiment of geological tectonic movement. The shape, slope and river Horton ratio of the watershed provide reliable data and theoretical support for the evolutionary characteristics of the land surface. In addition, hydrological watershed characteristic parameters have very important climatic and topographic significance. These parameters represent the comprehensive natural characteristics of a certain area and play a key role in the spatial distribution of hydrothermal conditions. Compared with topography and other factors, watershed characteristic parameters are more comprehensive in regulating climate. Therefore, the DEM-based watershed analysis can comprehensively reflect the spatial differentiation pattern of the underlying surface of a watershed according to the climatic and topographic characteristics, and the watershed scale is more representative.
3. Layer space analysis. In this study, the DEM-based watershed analysis method and land classification theory are combined to achieve land scale division. Then, using GIS spatial layer overlay technology, the land scale types represented by different dominant elements are analyzed step by step. According to the spatial land differentiation pattern, different scales of land can be obtained. The land elements, attributes, structure and function at different scales will differ, which is convenient for comprehensive analysis of the scale effect.

#### Land scale division system

In this study, the DEM-based watershed analysis method is combined with the landscape method and parameter method to determine the optimal land scale^45,46^. The naming of land scale types considers both scientific and practical applications. The advantage of the ‘subwatershed + soil + vegetation’ naming method used in this study is its intuitiveness. This approach can not only directly reflect the basis and process of land scale and type division but the naming method can also be adjusted and improved according to the actual universality and audience needs of follow-up research.

The main factors influencing the land scale in Fuping County are hydrology, soil and vegetation. The spatial overlay analysis function of ArcGIS was used to superimpose the watershed distribution map, soil type map and vegetation type map. In turn, the spatial analysis function was used to obtain the land scale types of Fuping County at different scales, which include the subwatershed scale, land chain scale and land segment scale (Table 5). The methods and steps of scale division also differ according to the research area and research purpose, and the detailed degree of scale level also differs.

**Table 5:** Scale division system of the spatial land continuum in Fuping County.

Level	Scale name	Leading factor	Dominant factor characterization indicators and standards	Number of types	Naming (example)
One-level	Subwatershed	Subwatershed (climate, topography, hydrology)	River watershed types: Dasha River Watershed, Gejiatai River Watershed, Pingyang River Watershed, Banyu River Watershed, Yaozi River Watershed, Upper Shahe River Watershed, Yanzhi River Watershed	7	Dasha River Watershed
Two-level	Land chain	Subwatershed, soil type	Soil types: Fluvo-aquic soil, Fluvo-cinnamon soil, Cinnamon soil, Calcareous cinnamon soil, Leaching cinnamon soil, Acid coarse bone soil, Calcareous coarse bone soil, Brown soil, Brown soil, Meadow soil	54	Types of acid coarse bone soil in the Dasha River Watershed
Three-level	Land segment	Subwatershed, soil type, vegetation type	Vegetation types: alpine meadow, coniferous and broad-leaved mixed forest, open forest shrub, shrub-grassland, crop	140	Types of acid coarse bone soil crops in the Dasha River Watershed

### Supplementary Information

Supplementary Information.

## Data Availability

The datasets used during the current study available from the corresponding author on reasonable request.
