# Supplementary material for: Land scale division and multifunctional evaluation for Fuping County, China, based on DEM-based watershed analysis
Source: Sci Rep. 2024 May 18;14:11384. doi: 10.1038/s41598-024-62252-3 (PMC11102491; doi:10.1038/s41598-024-62252-3)
Supplement: Supplementary file 1 — Supplementary Information. [file 41598_2024_62252_MOESM1_ESM.pdf]

I. CASA model-Production function

The production function to be evaluated in this study mainly refers to the production capacity of green plants, and the NPP of vegetation is selected as the evaluation index for production function. Here the NPP of vegetation refers to the total amount of organic dry matter produced by green plants per unit area and per unit time. The CASA model is used to estimate NPP based on the following formula:

$$NPP(x,t) = APAR(x,t) \times \varepsilon(x,t) \quad (1)$$

where the unit of NPP is ($gC/m^2/a$), APAR (x, t) refers to the photosynthetic effective radiation absorbed by pixel *x* in month *t* (gC/m^2/month), and ε (x, t) refers to the actual light energy utilization rate (gC/MJ) of pixel *x* in month *t*.

APAR (x, t) is calculated as follows:

$$APAR(x,t) = SOL(x,t) \times FPAR(x,t) \times 0.5 \quad (2)$$

where SOL (x, t) refers to the total solar radiation at pixel *x* in month *t* (MJ/m^2/month), FPAR (x, t) refers to the proportion of the incident photosynthetically effective radiation by vegetation, and the constant 0.5 refers to the proportion of solar radiation absorbed by vegetation, and

ε(x,t) is calculated as follows:

$$\varepsilon(x,t) = T_{\varepsilon 1}(x,t) \times T_{\varepsilon 2}(x,t) \times W_{\varepsilon}(x,t) \times \varepsilon_{max} \quad (3)$$

where $T_{\varepsilon 1}(x,t)$ and $T_{\varepsilon 2}(x,t)$ refer to the stress effect on light energy utilization efficiency under low and high temperature conditions, respectively, $W_{\varepsilon}(x,t)$ refers to the influence coefficient of water stress, which reflects the influence of water condition, and ε_{max} refers to the maximum light energy rate under ideal conditions (gC/MJ).

II. InVEST-Ecological function

1. Water conservation capacity

According to the principles of water balance and water circulation, water conservation capacity is calculated with the water production module of the InVEST model based on the combination of the annual average precipitation, annual average reference evapotranspiration, plant effective water content, plant root depth, and topography. The calculation is based on the following formula:

$$Y(x) = \left(1 - \frac{AET(x)}{P(x)}\right) \times P(x) \quad (4)$$

$$\frac{AET(x)}{P(x)} = 1 + \frac{PET(x)}{P(x)} - \left[1 + \left(\frac{PET(x)}{P(x)}\right)^{\omega}\right]^{\frac{1}{\omega}} \quad (5)$$

where Y(x) refers to the annual water production of the grid unit *x* (mm), AET(x) refers to the annual actual evaporation of the *x* (mm), P(x) refers to the annual precipitation of *x* (mm), PET(x) refers to the annual potential evaporation of *x* (mm), and ω refers to the non-physical parameter of the natural climate and soil property, whose value is set to 1.25.

2. Soil retention

The sediment transport ratio module in the InVEST model is used to calculate soil retention. The soil retention model is based on the general soil loss equation and the assumption of surface cover replacement. It comprehensively utilizes rainfall erosivity factor (R), soil erodibility factor (K), digital elevation model (DEM), terrain factor (LS), vegetation cover management factor (C), and soil and water conservation measure factor (P) to calculate soil retention. Soil retention amount can be expressed as the difference between the potential soil erosion amount and the actual soil erosion amount, and the specific calculation formula is as follows:

$$RKLS = R \times K \times LS \tag{6}$$

$$ULSE = R \times K \times LS \times C \times P \tag{7}$$

$$A_C = RKLS - ULSE \tag{8}$$

where RKLS refers to the potential soil erosion amount (t/hm^2/a), R refers to the rainfall erosion factor, K refers to the soil erodibility factor, LS refers to the length and slope factor, ULSE refers to the actual soil erosion amount (t/hm^2/a), C refers to the vegetation cover management factor, P refers to the soil and water conservation factor, and Ac refers to the soil conservation amount (t/hm^2/a).

A. Rainfall erosion factor R

Rainfall erosivity refers to the potential capacity of soil erosion caused by rainfall, which is an important factor in calculating soil retention as well as an objective dynamic indicators of soil separation and transportation caused by rainfall. In this study, the calculation of R is based on the formular described in the Guidelines for Calculating Soil Loss in Production and Construction Projects SL773-2018", which is as follows:

$$R = 0.067 \times {P_d}^{1.627} \tag{9}$$

where the unit of R is MJ·mm/(hm^2·h) and P_d refers to the average annual rainfall (mm).

B. Soil erodibility factor K

Soil erodibility refers to the difficulty degree of soil particles being separated and transported by water, which is mainly related to soil physical and chemical properties such as soil texture, soil structure, organic matter content, and soil permeability. It is calculated as follows:

$$K = \left(-0.01383 + 0.51575 K_{EPIC}\right) \times 0.1317 \tag{10}$$

$$K_{EPIC}=\left\{0.2+0.3exp\left[0.0256SAN\left(1-\frac{SIL}{100}\right)\right]\right\}\times\left(\frac{SIL}{CLA+SIL}\right)^{0.3}\times\left(1-\frac{0.25C}{C+exp(3.72-2.95C)}\right)\times\left(1-\frac{0.7\times(1-SAN)}{(1-SAN)+exp(22.9\times(1-SAN)-5.51)}\right) \tag{11}$$

where SIL refers to silt content (%), CLA refers to clay content (%), C refers to organic carbon content (%), and SAN refers to sand content (%).

C. Length and slope factor LS

The length and slope factor LS is also one of the factors affecting soil erosion, which reflects the impact of terrain and topography on soil erosion. Generally speaking, as the slope length increases, the inflow area of the slope surface increases, and the runoff, velocity, and hydraulic radius of downward flow also increase. LS is calculated as follows:

$$L=\left(\frac{\lambda}{22.1}\right)^m, \quad m=\begin{cases}0.2 & \theta\leq 1^\circ \\ 0.3 & 1^\circ<\theta\leq 3^\circ \\ 0.4 & 3^\circ<\theta\leq 5^\circ \\ 0.5 & \theta>5^\circ\end{cases} \tag{12}$$

$$S=\begin{cases}10.8\sin\theta+0.03 & \theta\leq 5^\circ \\ 16.8\sin\theta-0.05 & 5^\circ<\theta\leq 10^\circ \\ 21.9\sin\theta-0.96 & \theta>10^\circ\end{cases} \tag{13}$$

$$LS = L \times S \tag{14}$$

where L is the slope length factor, S is the slope factor, λ Refers to the slope length (m), constant 22.1 refers to the slope length of a standard unit plot, and m refers to the slope length index.

D. Vegetation cover management factor C

The vegetation coverage management factor refers to the ratio of soil loss under certain surface coverage and management measures to the soil loss on the control ground under the same conditions of timely tillage and continuous leisure, which is calculated as follows:

$$C=0.992exp\left(-0.0344\times\frac{NDVI-NDVI_{min}}{NDVI_{max}-NDVI_{min}}\right) \tag{15}$$

where $NDVI_{min}$ refers to the minimum NDVI value in the study area while $NDVI_{max}$ refers to the maximum NDVI value in the study area.

E. Soil and water conservation factor P

The soil and water conservation factor P refers to the ratio between the amount of soil loss protected by soil and water conservation methods and the amount of soil loss under the traditional condition that no protection measures are adopted, with a value ranging from 0 to 1, where 0 represents areas with good prevention and control measures and where erosion hardly occurs and 1 represents an area where no control measures are taken. According to the actual situation in the research area of this study, the P values of land types are shown in Table 1.

Table 1. P-value of soil and water conservation measures factors

Land use type		P value
Cultivated land	Slope 0-15°	0.15
	Slope 16°-25°	0.23
	Slope 26°-60°	0.75
	Slope >60°	1
Woodland		1
Grassland		1
Water area		0
Use of land in construction		0
Unused land		1

F. Carbon storage

The carbon storage module in InVEST is used to calculate carbon storage. The carbon storage model estimates carbon storage based on the land use cover map and the carbon density of its four carbon pools (aboveground biomass carbon pool, underground biomass carbon pool, litter carbon pool and soil carbon pool). The calculation is as follows:

$$C=\left(C_{above}+C_{below}+C_{dead}+C_{soil}\right)\times S \quad (16)$$

where C represents the total carbon storage (Mg), C_{above} refers to the aboveground biomass carbon density (Mg/hm^2), C_{below} refers to the underground biomass carbon density (Mg/hm^2), C_{dead} refers to litter carbon density (Mg/hm^2), and C_{soil} refers to soil carbon density per unit area (Mg/hm^2).

G. Habitat quality

The habitat quality module in the InVEST model is used to calculate habitat quality. Habitat quality refers to the ability of an ecosystem within a certain spatiotemporal range to provide suitable sustainable development and survival conditions for individuals and populations, which reflects the quality of biological habitats and thus serves as an important indicator of biodiversity. The habitat quality module, based on the sensitivity of landscape types and the intensity of external threats, starts from the perspective of biodiversity and determines the habitat quality by evaluating various habitat types and their degree of degradation. Habitat quality reflects ecological function, which is calculated as follows:

$$D_{xj}=\sum_{r=1}^{R}\sum_{y=1}^{Y_r}\left(\frac{\omega_r}{\sum_{r=1}^{R}\omega_r}\right)r_y i_{rxy}\beta_x S_{jr} \quad (17)$$

where r refers to the threat source, R refers to the total number of threat sources, y refers to the total number of grids in the threat source grid, and Y_r refers to the grid on the threat source r grid map, ω_r refers to the weight of the threat source, r_y refers to the threat factor value of grid y, i_{rxy} refers to the threat level of r_y to habitat grid $_x$, β_x refers to the accessibility level of grid x, and S_{jr} refers to the sensitivity of habitat type j to threat source r.

$$Q_{xj} = H_j\left(1 - \left(\frac{D_{xj}^z}{D_{xj}^z + K^z}\right)\right) \quad (18)$$

where Q_{xj} represents the habitat quality of the grid x of the j habitat, j represents the habitat type, x represents a different grid, Hj refers to habitat suitability, D_{xj} refers to the total threat level of habitat grid x, k refers to the semi-saturated parameter, which generally takes half of the maximum value of D_{xj}, and the z-value is generally 2.5.

Based on the actual situation of Fuping County and with reference to relevant literature and model parameter recommendations, this study selects cultivated land, urban and rural residential areas, and transportation land as habitat threat factors, and determines the impact weights and maximum impact distances of corresponding threat sources. Based on relevant research, the habitat suitability and sensitivity to threat factors required for the operation of the InVEST model were determined, which are summarized in Tables 2 and 3.

Table 2. Threat source parameters in the study area

Thread factor	Maximum impact distance /km	Weight	Type of spatial decay
Cultivated land	1	0.7	Linear
Urban and rural residential land	6	1	Exponential
Transportation land	3	0.6	Exponential

Table 3. Habitat suitability and sensitivity of different land use types to various threat sources in the study area

Land use type	Habitat suitability	Threat factor		
		Cultivated land	Urban and rural residential land	Transportation land
Cultivated land	0.4	0	0.3	0.2
Woodland	1	0.7	0.9	0.2
Grassland	0.6	0.6	0.7	0.2
Water area	0.9	0	0.9	0.2
Urban and rural residential land	0	0	0	0.8
Transportation land	0	0	0	0
Unused land	0.1	0.3	0.1	0.6
